# Validation of Subjective Well-Being Measures Using Item Response Theory

**DOI:** 10.3389/fpsyg.2019.03036

**Published:** 2020-01-22

**Authors:** Ali Al Nima, Kevin M. Cloninger, Björn N. Persson, Sverker Sikström, Danilo Garcia

**Affiliations:** ^1^Blekinge Center of Competence, Region Blekinge, Karlskrona, Sweden; ^2^Department of Psychology, University of Gothenburg, Gothenburg, Sweden; ^3^Anthropedia Foundation, St. Louis, MO, United States; ^4^Department of Psychology, University of Turku, Turku, Finland; ^5^Department of Psychology, Lund University, Lund, Sweden; ^6^Department of Behavioral Science and Learning, Linköping University, Linköping, Sweden

**Keywords:** Harmony in Life Scale, item response theory, Positive Affect Negative Affect Schedule, Satisfaction with Life Scale, subjective well-being

## Abstract

**Background:** Subjective well-being refers to the extent to which a person believes or feels that her life is going well. It is considered as one of the best available proxies for a broader, more canonical form of well-being. For over 30 years, one important distinction in the conceptualization of subjective well-being is the contrast between more affective evaluations of biological emotional reactions and more cognitive evaluations of one’s life in relation to a psychologically self-imposed ideal. More recently, researchers have suggested the addition of harmony in life, comprising behavioral evaluations of how one is doing in a social context. Since measures used to assess subjective well-being are self-reports, often validated only using Classical Test Theory, our aim was to focus on the psychometric properties of the measures using Item Response Theory.

**Method:** A total of 1000 participants responded to the Positive Affect Negative Affect Schedule. At random, half of the participants answered to the Satisfaction with Life Scale or to the Harmony in life Scale. First, we evaluate and provide enough evidence of unidimensionality for each scale. Next, we conducted graded response models to validate the psychometric properties of the subjective well-being scales.

**Results:** All scales showed varied frequency item distribution, high discrimination values (*Alphas*), and had different difficulty parameters (*Beta*) on each response options. For example, we identified items that respondents found difficult to endorse at the highest/lowest point of the scales (e.g., “Proud” for positive affect; item 5, “If I could live my life over, I would change almost nothing,” for life satisfaction; and item 3, “I am in harmony,” for harmony in life). In addition, all scales could cover a good portion of the range of subjective well-being (*Theta*): −2.50 to 2.30 for positive affect, −1.00 to 3.50 for negative affect, −2.40 to 2.50 for life satisfaction, and −2.40 to 2.50 for harmony in life. Importantly, for all scales, there were weak reliability for respondents with extreme latent scores of subjective well-being.

**Conclusion:** The affective component, especially low levels of negative affect, were less accurately measured, while both the cognitive and social component were covered to an equal degree. There was less reliability for respondents with extreme latent scores of subjective well-being. Thus, to improve reliability at the level of the scale, at the item level and at the level of the response scale for each item, we point out specific items that need to be modified or added. Moreover, the data presented here can be used as normative data for each of the subjective well-being constructs.

## Introduction

Subjective well-being refers to the extent to which a person believes or feels that his or her life is going well and is considered as one of the best available proxies for a broader, more canonical form of well-being (Diener et al., 2018). This line of research has led to important contributions with regard to physical, psychological, and social health (e.g., Cloninger, 2004; Eid and Larsen, 2008; Lyubomirsky, 2008; Diener et al., 2009; Kjell et al., 2013), thus, making subjective well-being a popular and interesting construct (OECD, 2013). For over 30 years, subjective well-being has been conceptualized as comprising affective and cognitive evaluations of one’s life (Diener, 1984; Diener et al., 2018). The affective component is conceptualized as affective evaluations of the emotions people experience in their daily lives, emotions such as, sadness, fear, anger, joy, etc. (cf. Watson et al., 1988). The cognitive component, on the other hand, is conceptualized as the way people evaluate their life as a whole in relation to a self-imposed ideal (Diener et al., 1985). Hence, one important distinction in the conceptualization of subjective well-being is the contrast between more affective evaluations that are obtained when asking about a person’s typical emotional experience and more cognitive, judgment-focused evaluations like life satisfaction (Diener et al., 2018).

Despite some debates regarding the best way to conceptualize and measure the affective component of subjective well-being (e.g., how frequent or how intensive positive and negative emotions are experienced, whether it is best to use experience sampling methods or recollections of experienced emotions), most researchers agree that the frequency of emotions, rather than how intensive emotions are experienced, is a better measure of the affective component (Diener et al., 2018). For instance, people who experience high levels of well-being experience intensive positive emotions very rarely (only 2.6% of the time); instead they feel contented or mildly happy very frequently (Diener and Diener, 1996; Diener and Seligman, 2002; Garcia and Erlandsson, 2011). Judgments of life satisfaction, on the other hand, have been the undisputed way to conceptualize the cognitive component of subjective well-being. More recently, however, researchers have suggested harmony in life as a complement or supplement to life satisfaction (Kjell et al., 2016; Kjell, 2018). Nevertheless, in contrast to the focus on a psychologically self-imposed ideal involved in evaluations of life satisfaction, harmony is the sense of balance and flexibility that an individual experience in relation to the world around her (Li, 2008a, b). Moreover, harmony is distinctive from life satisfaction, not only by means of relations to different constructs or psychometric properties of measures (i.e., the Satisfaction with Life Scale vs. the Harmony in Life Scale), but also through how people pursue harmony in their life (Kjell et al., 2016; Garcia et al., 2020b). Indeed, when people are asked to describe how they pursue harmony, the most frequent words they use are: *peace*, *balance*, *unity*, *agreement*, *calm*, *mediation*, *cooperation*, *tolerant*, *nature*, *forgiveness*, etc. (Kjell et al., 2016). In contrast, when asked to describe how they pursue life satisfaction, the most frequent words are: *job*, *money*, *achievement*, *education*, *success*, *wealth*, *house*, *gratification*, etc. (Kjell et al., 2016). Thus, conceptually, harmony is different from life satisfaction, not because it is a different cognitive component, but because the concept comprises behaviors and notions of a person being in balance, in agreement, or striving for equilibrium or unity with the world around her (Garcia et al., 2020b).

In sum, life satisfaction comprises cognitive evaluations of one’s life in relation to a psychologically self-imposed ideal (Diener et al., 1985), harmony comprises behavioral evaluations of how one is doing in a social context, and positive and negative affect comprises affective evaluations of biological emotional reactions. This is in line with the definition of health by the World Health Organization [WHO] (1946), in which health pertains not merely to the absence of disease or infirmity, but also to a state of physical, mental, and social well-being (see also Cloninger, 2004; VanderWeele, 2017). What is even more, it also corresponds to the biopsychosocial model, which is a scientific model that refers to a dynamic and complex interaction of physiological, psychological, and social factors that can both result in and contribute to health (Engel, 1977, 1980; Cloninger, 2004). Thus, we argue that the three subjective well-being components together are extremely important for our understanding of a complete biopsychosocial (cf. affect-cognition-behavior) model of subjective well-being (Garcia et al., 2020b). In this context, because most measures used to assess subjective well-being are self-reports, the cornerstone of research on a tentatively biopsychosocial model of subjective well-being should be to focus on the psychometric properties of the measures (Pavot, 2018). At a general level, the existing self-report measures exhibit strong psychometric properties including unidimensionality, high internal consistency, moderately strong test-retest reliability, and theoretically meaningful patterns of associations with other constructs and criteria (for reviews see Diener et al., 2009; Diener et al., 2013; for criticism regarding well-being measures see Brown et al., 2018). A clear majority of these analyses have implemented Classical Test Theory (CTT), which is a useful theory for understanding latent traits. To the best of our knowledge, there is little debate about the quality of these subjective well-being measures when researchers use these traditional methods (Diener et al., 2018; for criticism regarding well-being measures see Brown et al., 2018). However, evaluations of psychometric information of items and scales using CTT is dependent on the number of items and on the sample’s size and other features, so any changes of these features can strongly affect both item and the total psychometric properties of the scale (Oishi, 2007). For instance, more precise estimates of reliability coefficients and their confidence intervals are obtained in large sample sizes of at least 400 respondents (Charter, 1999), which is no so common when these measures have been tested (Leue and Lange, 2011). Moreover, using CTT researchers can only report a single value to represent the reliability of the scale that is under investigation. This is problematic because by using this type of analysis, researchers implicitly assume that the standard error of measurement is equal across all points in the continuum of the concept being measured (Oishi, 2007). Therefore, this type of analyses does not provide sufficient information at different points along the trait continuum (e.g., ranging from extremely satisfied with life to extremely unsatisfied with life). In other words, CTT does not yield detailed feedback about which items provide the most reliable information across range of true scores (Oishi, 2007). Instead, CTT considers a summated scale as a measure of the latent trait although it is created without any justification from the sum of item scores.

Indeed, as suggested by others, many of the advantages of modern methods (e.g., Item Response Theory, IRT) have been ignored when subjective well-being measures have been validated (Oishi, 2007). IRT is as relatively modern psychometric technique that overcomes some of these limitations. One of IRT’s biggest advantages is that we can determine how suitable items are to measure the latent traits, so it can increase reliable information and validity of the scale as a whole. The error and the reliable information obtained using IRT vary from one item to another and throughout the trait continuum of the scale, sometimes widely for one part of the scale compared with other parts (Oishi, 2007). In short, the aim of the present study is to apply IRT to evaluate existing well-validated measures^1^ that might constitute a tentative biopsychosocial model of subjective well-being (i.e., Positive Affect Negative Affect Schedule, Satisfaction with Life Scale, and Harmony in Life Scale). Next, we briefly present research regarding the psychometric properties of each of the measures.

### The Positive Affect Negative Affect Schedule

The Positive Affect Negative Affect Schedule was developed by Watson et al. (1988) as an attempt to provide better measures of positive and negative affect than contemporary measures at that time. These scales have been used in several studies to assess the affective or biological component of subjective well-being. Watson and colleagues started by selecting 60 adjectives representing affect from the factor analyses conducted by Zevon and Tellegen (1982). The selection criterion was that the adjectives were strongly correlated to one corresponding affect dimension but exhibited a weak correlation to the other. Throughout meticulous multiple rounds of selection and preliminary analyses, Watson et al. (1988) ended up with 10 items for each of the scales (see also Watson and Clark, 1994). That is, a total of 20 items consisting of 10 adjectives that measure positive affect (i.e., “Interested,” “Enthusiastic,” “Proud,” “Alert,” “Inspired,” “Determined,” “Attentive,” “Active,” “Excited,” and “Strong”) and 10 adjectives that measure negative affect (“Distressed,” “Upset,” “Guilty,” “Afraid,” “Hostile,” “Irritable,” “Ashamed,” “Nervous,” “Jittery,” and “Scared”) with a 5-point Likert (1 = *not at all*, 5 = *very much*). Watson et al. (1988) suggested that the orthogonal rotation of the factors is the best representation of positive and negative affect’s latent structure because of the opposing pleasant-unpleasant relationship in the factor loadings. The scales have shown high internal consistency in different studies — *Cronbach’s alphas* raging between 0.83 to 0.90 for positive affect and between 0.85 to 0.93 for negative affect (see Watson and Clark, 1994; Leue and Lange, 2011).

Nevertheless, researchers have reported a two-factor model with positive affect and negative affect as uncorrelated factors and correlated factors (e.g., Kercher, 1992; Krohne et al., 1996; Crocker, 1997; Mackinnon et al., 1999; Terraciano et al., 2003; Crawford and Henry, 2004) and also subfactors of positive affect and negative affect as uncorrelated and correlated first-order factors (e.g., Mehrabian, 1997; Killgore, 2000; Gaudreau et al., 2006). Moreover, validation studies (see Crawford and Henry, 2004) using structural equation modeling suggest that best-fitting models are achieved by specifying correlations between error in items closely related to each other in meaning: Distressed-Upset, Guilty-Ashamed, Scared-Afraid, Nervous-Jittery, Hostile-Irritable, Interested-Alert-Attentive, Excited-Enthusiastic-Inspired, Proud-Determined, and Strong-Active. Hence, these covariances suggest the possibility of item reduction without serious repercussions on the content domain or internal consistency reliability of the positive and negative affect scales (Thompson, 2007, 2017). Finally, despite a robust and impressive body of research, only a few studies have conducted IRT analyses to validate the Positive Affect Negative Affect Schedule (e.g., Pires et al., 2013 who showed, in a Brazilian sample, that the item Alert was the one with highest difficulty^2^ and worst fit statistics). Thus, IRT analyses are an important endeavor for the development of accurate and effective operationalization of the affective component of subjective well-being.

### The Satisfaction With Life Scale

The Satisfaction with Life Scale was originally developed by Diener et al. (1985) as a brief assessment of an individual’s general sense of satisfaction with her life (see also Pavot and Diener, 1993, 2008). It has been used in thousands of studies to assess the cognitive or psychological component of subjective well-being. Diener et al. (1985) developed the scale by first generating a pool of 48 items intended to reflect life satisfaction and well-being. Using factor analysis, they identified 10 items with high loadings (0.60 or above) on a common factor interpreted as global evaluations of a person’s life. After eliminating items with redundancies, Diener et al. further reduced the number of items to five (i.e., “In most ways my life is close to my ideal,” “The conditions of my life are excellent,” “I am satisfied with my life,” “So far I have gotten the important things I want in life,” and “If I could live my life over, I would change almost nothing”) with a 7-point Likert response scale (1 = *strongly disagree* to 7 = *strongly agree*).

The scale has high internal consistency as indicated by *Cronbach’s alphas* raging between 0.79 and 0.89 in some studies (Pavot and Diener, 1993), 0.87 (Adler and Fagley, 2005) and 0.86 (Steger et al., 2006) in other studies (for a meta-analysis see Vassar, 2008). Moreover, in the original article (Diener et al., 1985), the researchers showed that a principal-axis factor analysis on the Satisfaction with Life Scale resulted in a single factor solution, in which the single factor accounted for 66% of the variance of the scale. Despite the fact that the single factor solution has been replicated in several studies, the fifth item of the scale (“If I could live my life over, I would change almost nothing”) often shows lower factor loadings and item-total correlations than the first four items of the scale (e.g., Senécal et al., 2000). Pavot and Diener (2008) suggested that, because this specific item strongly implies a summary evaluation over past years, responses to it may involve a different cognitive recollection than the responses to the other items of the scale that imply a focus on the present (e.g., “The conditions of my life are excellent”) or a temporal summation (e.g., “In most ways my life is close to my ideal”). One way or the other, both CTT and the few studies using IRT methodology (e.g., Oishi, 2006) indicate that the fifth item of the Satisfaction with Life Scale is somewhat distinct from the other four items (Pavot and Diener, 2008). Since this item is highly correlated with the other four, however, it is not costume nor necessary or recommended to drop it from the measure (Pavot and Diener, 2008).

The few studies using IRT (Vittersø et al., 2005; Oishi, 2006) suggest that, in some cases, comparisons based on raw scores of the Satisfaction with Life Scale may be misleading. In one study, for example, although initial analyses showed no mean difference between Greenlanders and Norwegians, when IRT was applied, it was revealed that some Greenlanders were more prone to random responding, and to use extreme response categories. After controlling for these tendencies, Norwegians showed higher life satisfaction than Greenlanders, with the exception of a specific latent class of Greenlanders, who were in turn more satisfied than the Norwegian sample (Vittersø et al., 2005).

### The Harmony in Life Scale

The Harmony in Life Scale was developed by Kjell et al. (2016) who suggested that focusing solely on life satisfaction as the cognitive component of subjective well-being is problematic since individuals think about their life in various ways (cf. Delle Fave et al., 2011). Based on a literature review of global contexts, such as, lifestyle, surroundings, conditions, environment, society and the world, Kjell et al. (2016) generated 29 items that included essential key concepts such as harmony, being attuned, fitting in, acceptance, adaptation, adjustment, and peace of mind. These items were evaluated by 5 experts within psychological research who were presented with a review of the aims and theories underlying the scale and asked to rate each item based on relevance (cf. Davis, 1992). Based on these evaluations the final numbers of items amounted to 15. The 15 items were randomly presented, with the same instructions and Likert Scale as the Satisfaction with Life Scale, to 476 respondents. Kjell et al. (2016) used an exploratory factor analysis based on maximum likelihood and promax rotation to explore the factor structure of the scale. The analysis revealed a clear single factor model with the total eigenvalue of 9.40 explaining 62.64%, while the factor loadings for the 15 items ranged from 0.56 to 0.86. The researchers eliminated redundant items and chose five items (i.e., “My lifestyle allows me to be in harmony,” “Most aspects of my life are in balance,” “I am in harmony,” “I accept the various conditions of my life,” and “I fit well with my surroundings”) that they found relevant to their theoretical framework and with factor loadings ranging from 0.73 to 0.86 (see also Singh et al., 2016 for factor loadings ranging from 0.75 to 0.90) and a *Cronbach’s alpha* of 0.90 (see also Garcia et al., 2014 for a *Cronbach’s alpha* of 0.91, Kjell et al., 2019 for *Cronbach’s alphas* between 0.89 and 0.95, and Singh et al., 2016 for *Cronbach’s alphas* between 0.83 and 0.87).

In a second study in the same article (Time 1 *n*_1_ = 787 and Time 2 *n*_2_ = 545), Kjell et al. (2016) showed that the Harmony in Life Scale had good test-retest reliability (*r* = 0.77) and that it correlated as expected to other well-being related scales, such as, the Satisfaction with Life Scale (*r* = 0.76) and the Subjective Happiness Scale (*r* = 0.67). Interestingly, CTT analyses showed that despite a strong correlation between life satisfaction and harmony in life, the two-factor models, rather than single factor models, were considerable better at both Time 1 [χ*^2^*(34) = 191.70, *p* < 0.001; *CFI* = 0.97; *RMSEA* = 0.08] and Time 2 [χ*^2^*(34) = 120.72, *p* < 0.001; *CFI* = 0.98; *RMSEA* = 0.07]. Moreover, to the best of our knowledge, the Harmony in Life Scale has only been used in three published articles besides the original study (i.e., Garcia et al., 2014; Singh et al., 2016; Kjell et al., 2019) and no study has used IRT as a method for psychometric testing.

### Item Response Theory and the Present Study

IRT is a family of psychometric methods for analysis of items, item responses as well as whole scale properties. The basic premise of IRT is that the probability of a response is a function of an underlying trait, continuum (latent dimension) or ability that is denoted by Theta (θ). Theta represents a person’s true latent trait (e.g., subjective well-being), which has been standardized to follow standard normal distribution with a range from −3.00 to 3.00, with 0.00 representing the average score (Baker, 2001). The primary goal of using IRT is to validate and modify existing scales that measure how much of a latent trait one person has, in this case positive affect, negative affect, life satisfaction, and harmony. For example, IRT can be applied to investigate which items that haven’t enough reliable information about the construct and which parts of that construct that the items don’t measure. IRT analyses can also differentiate items’ properties (e.g., discrimination and difficulty) among individuals across a much wider range of the construct at hand. If the analyses show that there is such a problem with some items, the researcher can remove/modify those items or add new items that help to measure these parts of the construct, thus, providing information that can differentiate people across a much greater range of the latent trait and increases the validity of the whole scale (Oishi, 2007). Also, IRT analyses might help clinicians to understand patients’ behavior regarding a difficult or easy item, which might be helpful for intervention as well as for normative data (Pires et al., 2013).

The items of the scales used to measure subjective well-being (i.e., Positive Affect Negative Affect Schedule, the Satisfaction with Life Scale, and the Harmony in Life Scale) are ordinal and scored on Likert scales, so the appropriate IRT model for them is a graded response model (GRM). In GRM each item has its own estimated difficulty scores or threshold parameter (i.e., Beta, β) that represents the underlying latent trait for each response for each person. More specifically, Beta represents the level of the underlying trait at which the next response option has 50% chance of being endorsed. Moreover, each item in GRM has also its discrimination parameter (i.e., Alpha, α) which reflects how well the items discriminate between different levels of the latent trait. Moreover, Alpha is used to reflect how strongly an item is related with this latent trait, so it can be considered roughly equivalent to factor loadings used in CTT. The discrimination parameter values can be from −∞ to +∞, but values are typically at about 0 to +2.50. Here, item discrimination values of 0.01–0.34 are considered very low; 0.34–0.64 low; 0.65–1.34 moderate; 1.35–1.69 high; and 1.70 and above very high (Baker, 2001). It is usually recommended to delete the items with negative value, because this might suggest that something is wrong with the item since it indicates that the probability of a correct response decreases while the ability increases (Baker, 2001).

In order to use IRT models, there are some basic assumptions regarding unidimensionality, local independence, monotonicity (shape of curve) and differential item functioning (DIF). Unidimensionality states that the set of items in the questionnaire/test are expected to load on only one latent factor to explain the item response patterns. This is tested using factor analysis. Local independence means that the latent trait score explains most of the variance of participants’ responses to the items in the scale. This is tested by verifying that the residuals for each item is not significantly correlated to the residuals of any other item in the scale. Monotonicity refers to item characteristics that reflect the true relationship between the person’s latent trait score and the participant’s actual response to the item. In other words, IRT models assume that the levels of the person’s latent trait increase, as a monotonical function, as the probability to choosing the answer in each item that represents the participants actual level of the trait increases. DIF is applied to investigate so that the differences regarding the responses to each item does not vary across different groups (e.g., men and women).

Again, more sophisticated statistical techniques based on IRT (e.g., techniques described above that address the properties of the whole scale, items, and item responses at the population and subpopulation level) seem to present a promising way forward for the measurement of subjective well-being (Oishi, 2007; OECD, 2013). Our aim was to investigate, using IRT methods, the psychometric properties of the two instruments that are commonly used to measure the affective (or biological) and cognitive (or psychological) components of subjective well-being (i.e., the Positive Affect Negative Affect Schedule and the Satisfaction with Life Scale) along a new measure, tentatively suggested to measure the behavioral (or social) component (i.e., the Harmony in Life Scale). These measures are not only the most common when measuring the different components, but as reviewed in the introduction, they have good psychometric properties and are unidimensional in nature as analyzed using CTT in past research. Unidimensionality, is by the way, an important assumption for IRT analyses. To the best of our knowledge, this is the first study to examine these three subjective well-being instruments in the same study using IRT.

## Materials and Methods

### Ethics Statement

Ethics approval was not required at the time the research was conducted as per national regulations. The consent of the participants was obtained by virtue of survey completion after they were provided with all relevant information about the research (e.g., anonymity).

### Participants and Data Collection Procedure

The participants were recruited through Amazon’s Mechanical Turk^3^ ^,4^. All participants originated from the United States and spoke English as their first language. Participants were informed that the survey was voluntary, anonymous, that they could terminate the survey at any time and that those who accepted would receive $0.50 as compensation for their participation. We added two control questions to the survey, to control for automatic responses (e.g., “This is a control question, please answer “either agree or disagree”). The final sample, after taking away those who responded erroneously to one or both of the control questions (*n* = 100, 9.09% of all respondents) consisted of 1000 participants (404 males and 596 females), including two who did not report their age (age *mean* for 998 participants = 34.22, *SD* = 12.73, range from 18 to 74). All 1000 participants responded to the Positive Affect Negative Affect Schedule. However, since the instructions, the format, and response scale of the Satisfaction with Life Scale and the Harmony in life Scale are exactly the same, participants were randomly presented with the Satisfaction with Life Scale (age *mean* for 498 participants = 34.08, *SD* = 12.55, range from 18 to 74; male = 217 and female = 283) or the Harmony in Life Scale among the participants (age *mean* for 500 participants = 34.36, *SD* = 12.92, range from 18 to 73; male = 187 and female = 313). This was done in order to avoid any likeness between the scales to influence participants’ responses.

### Measures

The Positive Affect Negative Affect Schedule (Watson et al., 1988) measures a person’s experience of positive and negative affect. The respondents are asked to estimate and rate to which extent they have felt 10 positive (e.g., “Attentive”) and 10 negative (e.g., “Hostile”) feelings and moods during the last week on a five-point scale (1 = *very slightly or not at all*, 5 = *extremely*).

The Satisfaction with Life Scale (Diener et al., 1985) measures individuals’ global cognitive judgments of their life as a whole in relation to a self-imposed ideal using five items (e.g., “In most ways my life is close to my ideal”) and a seven-point Likert scale (1 = *strongly disagree*, 7 = *strongly agree*).

The Harmony in Life Scale (Kjell et al., 2016) assess a person’s global sense of harmony in life and consists of five statements (e.g., “My lifestyle allows me to be in harmony”) for which respondents are asked to indicate degree of agreement on a seven-point Likert scale (1 = *strongly disagree*, 7 = *strongly agree*).

### Statistical Procedure

We used the following software to analyze the data: STATA version 14, R, SPSS version 24, and AMOS version 24. First, we used exploratory factor analysis (EFA) and confirmatory factor analysis (CFA) to replicate past evidence showing that the correlation among items in each measure is explained by only a single latent trait (i.e., showing unidimensional factor structures). The lack of unidimensionality, for instance, might lead to biased results regarding IRT parameter estimates^5^. For each of the subjective well-being measures, EFA showed that the scree plot of eigenvalues suggested a single latent factor. The first eigenvalues of each scale (3.56 for life satisfaction, 3.74 for harmony in life, 5.08 for positive affect, and 1.05 for negative affect) were much greater than the others, which were less than 1.06. The ratio of the first to the second eigenvalue was greater than 5.00. Hence, for all scales there is evidence of unidimensionality (cf. Sattelmayer et al., 2017). Item loadings ranged from 0.63 to 0.80 for positive affect, 0.63 to 0.80 for negative affect, 0.74 to 0.90 for life satisfaction, and 0.79 to 0.91 for harmony in life.

The basic single factor CFA model for positive affect showed that the *chi-square* value was significant (χ^2^ = 443.59, *df* = 35, *p* < 0.001), the *goodness of fit index* was 0.91, the *incremental fit index* was 0.91, and the *Root Mean Square Error of Approximation* fit statistic was slightly outside the acceptable rang 0.108 (for more details see Supplementary Figure S1). After one modification, a path between the error measurement for Alert-Attentive, the *chi-square* value was lower, but still significant (χ*^2^* = 307.55, *df* = 34, *p* < 0.001). Nevertheless, after this modification, all other fit indexes were acceptable (the *goodness of fit index* was 0.94, the *incremental fit index* was 0.94, and the *Root Mean Square Error of Approximation* fit statistic that was 0.09). All factor loadings were significant at *p* < 001 (Supplementary Figures S1, S2).

The basic single factor CFA model for negative affect showed that the *chi-square* value was significant (χ*^2^* = 1055.38, *df* = 35, *p* < 0.001). Fit indexes were slightly outside the traditional acceptable range: the *goodness of fit index* was 0.80, the *incremental fit index* was 0.82, and the *Root Mean Square Error of Approximation* fit statistic that was 0.17 (for more details see Supplementary Figure S3). After three modifications, paths between the error measurements for Guilty-Ashamed, Hostile-Irritable, and Afraid-Scared, the *chi-square* value was lower but still significant (χ*^2^* = 438.53, *df* = 32, *p* < 0.001). Nevertheless, after these modifications, all other fit indexes were acceptable (the *goodness of fit index* was 0.91, the *incremental fit index* was 0.93, and the *Root Mean Square Error of Approximation* fit statistic that was 0.11). All factor loadings were significant at *p* < 001 (for more details see Supplementary Figures S3, S4).

The basic single factor CFA model for life satisfaction fitted well (Supplementary Figure S5). The results showed that the *chi-square* value was not significant (χ*^2^* = 10.14, *df* = 5, *p* = 0.07), the *goodness of fit index* was 0.99, the *incremental fit index* was 1.00, and the *Root Mean Square Error of Approximation* fit statistic that was 0.04. Thus, indicating that the model fit was acceptable (cf. Bollen, 1989; Browne and Cudeck, 1993). All factor loadings were significant at *p* < 001.

The basic single factor CFA model for harmony in life fitted also well (Supplementary Figure S6). The results showed that the *chi-square* value was significant (χ*^2^* = 31.68, *df* = 5, *p* < 0.001). The *goodness of fit index* was 0.98, the *incremental fit index* was 0.99, and the *Root Mean Square Error of Approximation* fit statistic that was 0.10. That is, all indexes indicated that the model fit was acceptable. All factor loadings were significant at *p* < 001.

Previous research suggests that fit indexes that are slightly outside the traditional acceptable range can be considered as sufficiently unidimensional for further IRT analysis (Cook et al., 2009; Stepp et al., 2012). In addition, although significant for some of the models, the *chi-square* statistic is heavily influenced by sample size (Kline, 2010), with larger samples leading to a larger value and therefore, a larger likelihood of being significant. Thus, given the results of the scree plot of eigenvalues, eigenvalues, ratios, item loadings and the results of the CFA, we considered that our results provide sufficient evidence of unidimensionality of single latent trait for each one of these four main measures of a biopsychosocial model of subjective well-being.

Regarding local independence, our analyses showed that, for all scales, the residuals (i.e., differences between the individuals’ observed scores and their respective predicted scores) of almost each paired correlation were significant. That is, most of the items can be considered as locally dependent and that our data had a tendency for multidimensionality. See Supplementary Tables S2a,b for the details. Result regarding Monotonicity indicated that the response function of the probability of getting correct response of each item of each scale increased when the person’s latent trait level increased. See Supplementary Table S3 and Supplementary Figure S7 for the details. The result exhibited uniform Differential Item Function (DIF) for each item in SWLS across gender. This indicated that the ability of a person to answer does not change due to gender characteristics. See Supplementary Figure S8 for the details.

We tested the item fit statistic using the Orlando–Thissen–Bjorner *item fit S-*χ*2 statistic* to determine absolute fit of the model to each item. Regarding S-χ2 statistic, a value that is not significant indicates that the model adequately fits an item. The result indicated that 25 items were adequately fit, while four items were statistically significant at *p* < 0.05 and one item at *p* < 0.01. The S-χ2 statistic is sensitive and influenced by sample size, test length and multiple comparisons, with larger samples, small test length and multiple comparisons leading to a larger value and therefore, a larger likelihood of being significant (*Type I error*). In other words, these five valid items were falsely identified as mis-fitting when in fact the model fitted the data/items, so the root mean square error of approximation (RMSEA) was used but it was based on the S-χ2 statistic (RMSEA S-χ2). Traditional cut-offs for RMSEA tend to be RMSEA ≤ 0.08 to determine absolute fit of the model to each item. The result exhibited that the largest value of RMSEA S-χ2 was 0.03, so this result indicated an adequate item-level model-data fit. Nevertheless, we applied the Benjamini–Hochberg criterion for *p*-value adjustment (Benjamini and Hochberg, 1995). Three items (“Scared,” “My lifestyle allows me to be in harmony,” and “I fit in well with my surroundings”) were still significant after correction (see Supplementary Table S4). We checked these items’ information, difficulty, and discrimination parameter in order to decide whether they needed to be excluded from the analyses. Since these three items provided with reliable information, discrimination and difficulty, along good properties overall (see for example analyses regarding monotonicity), we decided to keep them. For example, the item “Scared,” was still significant after correction, but this item had good reliable information, high discrimination parameter 3.49 and difficulty parameters between 0.26 and 1.94, which are even better values that some of the items that were not significant after correction. See Supplementary Table S4 for the details.

#### Comparisons Among GRM, RSM and PCM

In order to determine the most appropriate IRT model to our data, we compared the model we chose, GRM, with both *Rating Scale Model* (RSM), which is for ordinal responses to items that share the same rating scale structure, and *Partial Credit Model* (PCM), which is for ordinal responses to item that have its own rating scale structure. We used three fit indices to evaluate model fit: *Log-likelihood*, *Bayesian information criterion* (BIC) and *Akaike information criterion* (AIC). The result showed that GRM was preferable. See Supplementary Table S1 for the details.

## Results

### IRT Analyses of the Positive Affect Negative Affect Schedule

#### Positive Affect

**FIGURE 1:**
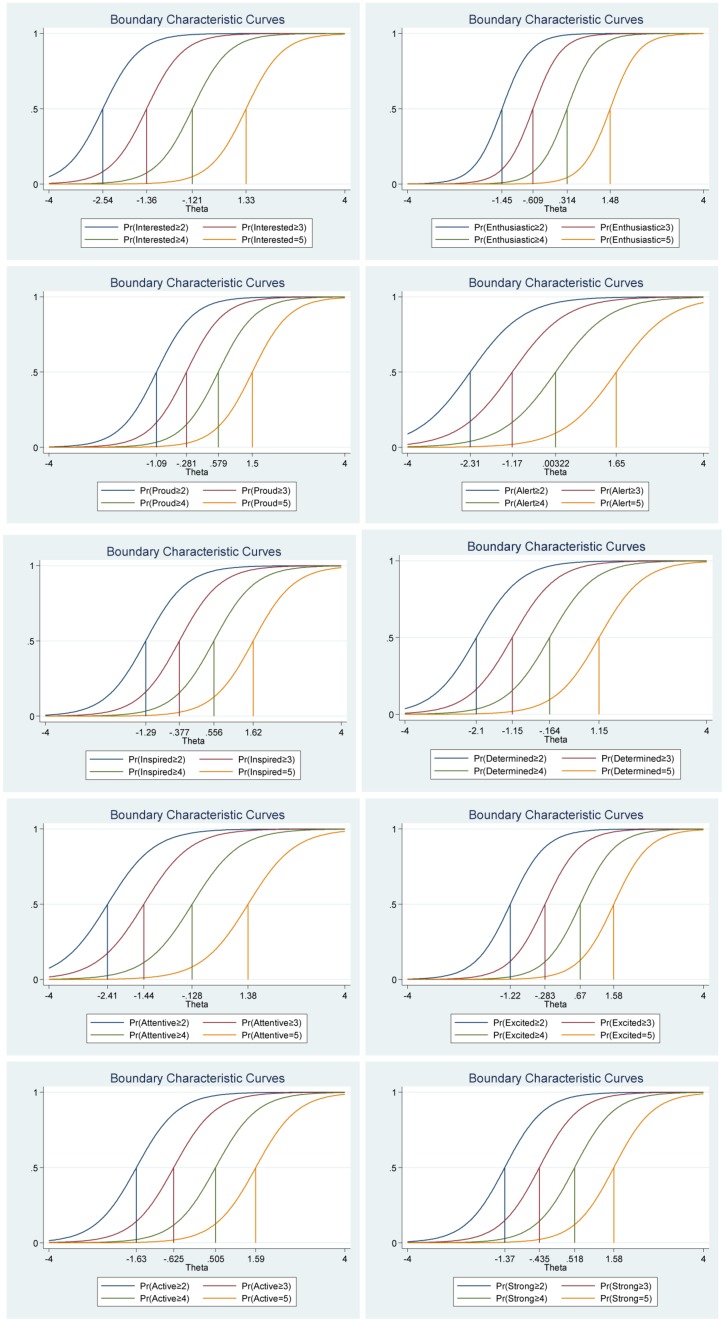
Boundary characteristic curves for each item of the positive affect scale of the Positive Affect Negative Affect Schedule (*N* = 1000).

We found that the frequency distributions for each of the items in the positive affect scale were different (see Table 1), for example, for the item “Determined” 20.80% of the participants reported the highest levels (5 = *extremely*) compared with the item “Enthusiastic” for which only 10.30% of the participants reported the highest levels (5 = *extremely*). The item “Enthusiastic” was more difficult, explained through the proportion of participants choosing the highest point of the scale, than the item “Determined.” This is important, if the items vary in their difficulty, the correlations among items would be small. Moreover, in this analysis each item gets its own discrimination/slope (Alpha) and own ‘location’ parameter (Beta); the differences between categories around that location are not equal across items (see Table 2 and Figure 1). Regarding item discrimination, all items had high discrimination values (Alphas from 1.37 to 2.65) and demonstrated a steeper slope, which indicates that the items can differentiate well between persons with high and low levels of the latent score of positive affect (see Table 2 and Figure 1). Regarding the estimated threshold/difficulty parameter (Beta) for the positive affect scale were between -2.54 and 1.65 (see Table 2). The item “Alert” had the highest estimated difficulty parameter on response 5 (β = 1.65) and the item “Interested” had the lowest estimated difficulty parameter on response 1 (β = −2.54). To understand the difficulty parameter, let’s exemplify with the first item, “Interested.” A respondent with −2.54 in positive affect has a 50% chance of answering 1 (*very slightly or not at all*), versus greater or equal chance of answering 2 (i.e., responses 2, 3, 4, or 5). A respondent with −1.36 in positive affect has a 50% chance of answering 1 or 2, rather than greater or equal chance of answering 3 (i.e., responses 3, 4, or 5). A person with 1.33 in positive affect has a 50% chance of picking response 5 (*extremely*), rather than less or equal chance of answering 4 (i.e., responses 1, 2, 3, or 4).

**TABLE 1:** The frequency distributions of the positive affect scale of the Positive Affect Negative Affect Schedule (*N* = 1000).

Item	Points in the Likert Scale				
	1	2	3	4	5
**Interested**					
Frequency	32	113	309	396	150
Percent	3.20	11.30	30.90	39.60	15.00
Cumulating	3.20	14.50	45.40	85.00	100.00
**Enthusiastic**					
Frequency	115	183	300	299	103
Percent	11.50	18.30	30.00	29.90	10.30
Cumulating	11.50	29.80	59.80	89.70	100.00
**Proud**					
Frequency	199	205	263	209	124
Percent	19.90	20.50	26.30	20.90	12.40
Cumulating	19.90	40.40	66.70	87.60	100.00
**Alert**					
Frequency	79	152	273	347	149
Percent	7.90	15.20	27.30	34.70	14.90
Cumulating	7.90	23.10	50.40	85.10	100.00
**Inspired**					
Frequency	175	212	269	227	117
Percent	17.50	21.20	26.90	22.70	11.70
Cumulating	17.50	38.70	65.60	88.30	100.00
**Determined**					
Frequency	71	125	244	352	208
Percent	7.10	12.50	24.40	35.20	20.80
Cumulating	7.10	19.60	44.00	79.20	100.00
**Attentive**					
Frequency	55	101	301	373	170
Percent	5.50	10.10	30.10	37.30	17.00
Cumulating	5.50	15.60	45.70	83.00	100.00
**Active**					
Frequency	119	198	328	233	122
Percent	11.90	19.80	32.80	23.30	12.20
Cumulating	11.90	31.70	64.50	87.80	100.00
**Excited**					
Frequency	169	243	290	188	110
Percent	16.90	24.30	29.00	18.80	11.00
Cumulating	16.90	41.20	70.20	89.00	100.00
**Strong**					
Frequency	154	214	281	231	120
Percent	15.40	21.40	28.10	23.10	12.00
Cumulating	15.40	36.80	64.90	88.00	100.00

**TABLE 2:** Item response analysis of the positive affect scale in the Positive Affect Negative Affect Schedule (*N* = 1000).

Item	Coef.	*SE*	*Z*	*P*	95% CI	
**Interested**						
Discrimination	2.03	0.12	17.58	0.00	1.81	2.26
Difficulty						
≥2	−2.54	0.14	−18.54	0.00	−2.81	−2.28
≥3	−1.36	0.08	−17.62	0.00	−1.51	−1.21
≥4	−0.12	0.05	−2.40	0.02	−0.22	−0.02
= 5	1.33	0.08	17.39	0.00	1.18	1.48
**Enthusiastic**						
Discrimination	2.65	0.15	17.56	0.00	2.35	2.94
Difficulty						
≥2	−1.45	0.07	−19.72	0.00	−1.59	−1.30
≥3	−0.61	0.05	−11.89	0.00	−0.71	−0.51
≥4	0.31	0.05	6.72	0.00	0.22	0.41
= 5	1.48	0.07	19.81	0.00	1.33	1.63
**Proud**						
Discrimination	2.00	0.11	17.43	0.00	1.77	2.22
Difficulty						
≥2	−1.09	0.07	−15.52	0.00	−1.23	−0.95
≥3	−0.28	0.05	−5.39	0.00	−0.38	−0.18
≥4	0.58	0.06	10.52	0.00	0.47	0.69
= 5	1.50	0.08	17.78	0.00	1.33	1.66
**Alert**						
Discrimination	1.37	0.09	15.83	0.00	1.20	1.54
Difficulty						
≥2	−2.31	0.14	−16.06	0.00	−2.59	−2.03
≥3	−1.17	0.09	−13.63	0.00	−1.34	−1.00
≥4	0.00	0.06	0.05	0.96	−0.12	0.12
=5	1.65	0.11	15.20	0.00	1.43	1.86
**Inspired**						
Discrimination	1.81	0.11	17.20	0.00	1.60	2.02
Difficulty						
≥2	−1.29	0.08	−16.14	0.00	−1.44	−1.13
≥3	−0.38	0.06	−6.79	0.00	−0.49	−0.27
≥4	0.56	0.06	9.69	0.00	0.44	0.67
=5	1.62	0.09	17.40	0.00	1.44	1.80
**Determined**						
Discrimination	1.71	0.10	16.90	0.00	1.51	1.91
Difficulty						
≥2	−2.10	0.12	−17.57	0.00	−2.34	−1.87
≥3	−1.15	0.08	−15.00	0.00	−1.31	−1.00
≥4	−0.16	0.05	−3.00	0.00	−0.27	−0.06
=5	1.15	0.08	15.06	0.00	1.00	1.30
**Attentive**						
Discrimination	1.58	0.10	16.35	0.00	1.39	1.77
Difficulty						
≥2	−2.41	0.14	−16.92	0.00	−2.69	−2.14
≥3	−1.44	0.09	−15.67	0.00	−1.62	−1.26
≥4	−0.13	0.06	−2.26	0.02	−0.24	−0.02
=5	1.38	0.09	15.46	0.00	1.21	1.56
**Active**						
Discrimination	1.78	0.10	17.29	0.00	1.57	1.98
Difficulty						
≥2	−1.63	0.09	−17.27	0.00	−1.82	−1.45
≥3	−0.62	0.06	−10.36	0.00	−0.74	−0.51
≥4	0.51	0.06	8.81	0.00	0.39	0.62
=5	1.59	0.09	17.18	0.00	1.41	1.78
**Excited**						
Discrimination	2.11	0.12	17.61	0.00	1.87	2.34
Difficulty						
≥2	−1.22	0.07	−16.82	0.00	−1.37	−1.08
≥3	−0.28	0.05	−5.57	0.00	−0.38	−0.18
≥4	0.67	0.06	11.98	0.00	0.56	0.78
=5	1.58	0.08	18.66	0.00	1.41	1.74
**Strong**						
Discrimination	1.87	0.11	17.27	0.00	1.65	2.08
Difficulty						
≥2	−1.37	0.08	−16.69	0.00	−1.53	−1.21
≥3	−0.44	0.06	−7.86	0.00	−0.54	−0.33
≥4	0.52	0.06	9.24	0.00	0.41	0.63
=5	1.58	0.09	17.58	0.00	1.40	1.75

Furthermore, the differences between categories around difficulty parameters (Beta) are not equal across items. That is, for each item a response of, for example, 5 (*extremely*) was treated differently: β = 1.65 for item “Alert” while it was 1.15 for item “Determined.” Moreover, the differences in difficulty varied within each item (i.e., distances between responses for each item). For example, for the item “Interested” (see Table 2), the difference between ≥2 and ≥3 is −2.54 – (−1.36) = −1.18, while the difference between ≥3 and ≥4 is −1.36 – (−0.12) = −1.24. Thus, participants’ total score of positive affect will differ from totals scores using CTT, where differences are treated as equal and added without further justification (for more details see Table 2 and Figure 1).

**FIGURE 2:**
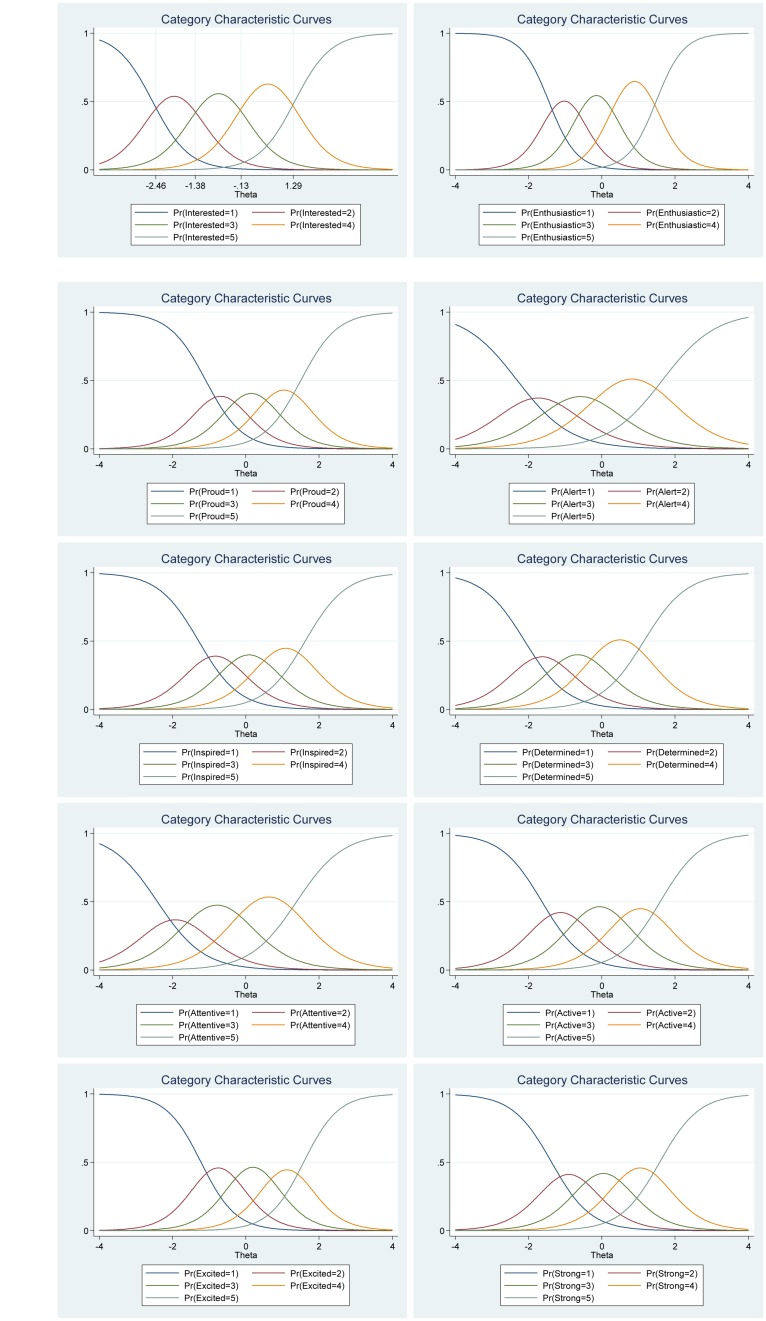
Category characteristic curves for the items in the positive affect scale of the Positive Affect Negative Affect Schedule (*N* = 1000).

The graph regarding category characteristic curves (Figure 2) gives information about the relationship between the level of the participants’ positive affect (i.e., the latent trait) and the probability of responding to specific points in the scale for each item, respectively. The graphs show the location where the next category becomes more likely (not 50%), that is, the points where the adjacent categories cross represent transitions from one response point to the next. For example, for the item “Interested,” participants with positive affect (latent trait) levels below −2.46 are more likely to respond 1 (*very slightly or not at all*) while the participants with positive affect levels between −2.46 and −1.38 are most likely to respond 2, and so on. Moreover, the probability of option 1 and 5 for this item are about equal and very high (For more details see Figure 2).

**FIGURE 3:**
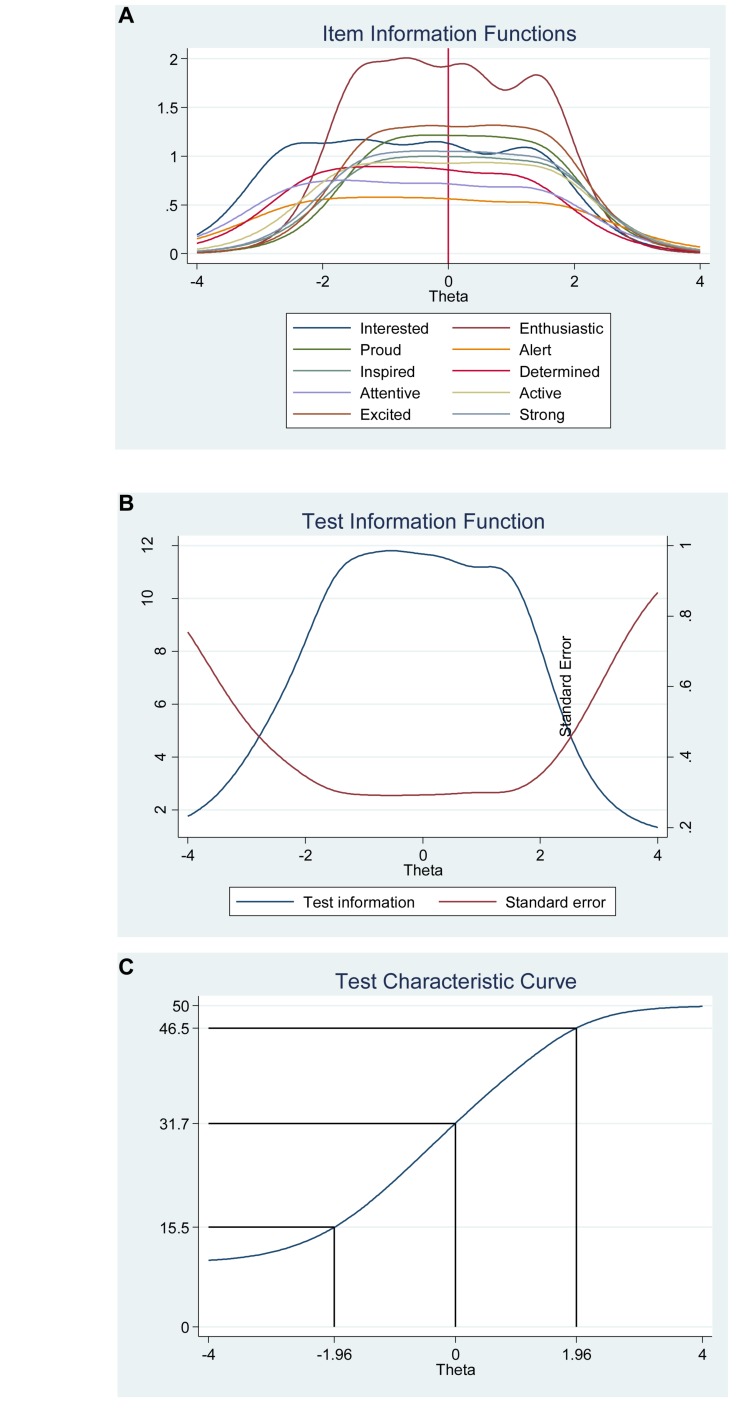
Items information function graphs for graded response and with vertical line at θ = 0 **(A)** and information and standard error graph for graded response **(B)** and test characteristic curve **(C)** for the whole positive affect scale of the Positive Affect Negative Affect Schedule (*N* = 1000).

We also investigated the item information function (see Figure 3A) for each item to see how much information each item provides as estimated by their location on the continuum (i.e., difficulty parameter) for the latent factor of positive affect and to investigate what level of the continuum each item has most or least information or reliability. In other words, the item information function reflects the properties of each item in terms of both its difficulty (Beta) and discrimination (Alpha) index. Moreover, this analysis helped us to evaluate where additional items would be useful to develop the scale. For instance, the items “Enthusiastic” and “Excited” had the highest discrimination estimates and seem to provide more information than the remaining items, while the items “Alert” and “Attentive” provide lesser information. In general, the items cover the distribution of the true range of positive affect (Theta, θ) from low (−2.50) up to high (2.30). Moreover, we show that we get reliable information at θ = 0 (vertical red line in Figure 3A) at about 1.90 from the item “Enthusiastic,” at about 1.30 from the item “Excited,” at about 1.20 from the item “Proud,” at about 1.10 from the item “Interested,” at about 1.05 from item “Strong,” and so on.

Moreover, the 10 items together provide a lot of information to measure positive affect among participants that vary within range −2.50 up to about 2.30 (Theta) of the level of the scale of positive affect (see Figure 3B, test information function and the standard error, that is, measurement error). This means that the positive affect scale has good reliability and small standard error in this range. The test information highest level is located at −0.50 (Theta), thus indicating that this score has the smallest standard error and provides the most information of the scale. However, there is almost no reliable information below -3.50 and above 3.50 (Theta) and the standard error increases quickly for both smaller and larger Theta values. The reliability for different levels of positive affect are shown in Table 3. These results showed that the scale’s reliability is very strong (between 0.88 to 0.91) at θ = −2.00, θ = −1.00, θ = 0.00, θ = 1.00, and θ = 2.00, that reliability is good (0.75) at θ = −3.00, but that reliability is week (0.64) at θ = 3.00.

**TABLE 3:** Reliability of the fitted graded response IRT model of the positive and negative affect scales of the Positive Affect Negative Affect Schedule (*N* = 1000).

Theta	Positive Affect			Negative Affect		
	Test Information Function	Test Information Function-*SE*	Reliability IRT GRM	Test Information Function	Test Information Function-*SE*	Reliability IRT GRM
−3.00	4.00	0.50	0.75	1.11	0.95	0.10
−2.00	8.37	0.35	0.88	1.86	0.73	0.46
−1.00	11.66	0.29	0.91	6.33	0.40	0.84
0.00	11.68	0.29	0.91	14.96	0.26	0.93
1.00	11.19	0.30	0.91	18.89	0.23	0.95
2.00	8.17	0.35	0.88	17.69	0.24	0.94
3.00	2.80	0.60	0.64	6.80	0.38	0.85

**Cronbach’s alpha* for the whole scale using CTT were 0.90 for the positive affect scale and 0.91 for negative affect scale.*

Figure 3C shows the test characteristic curve for the whole scale, which indicates the expected score against the latent trait (i.e., positive affect) as a sum of the probabilities. Since the positive affect scale of the Positive Affect Negative Affect Schedule has 10 items with a five-point Likert scale (1 = *very slightly or not at all*, 5 = e*xtremely*), the expected scores are between 10 and 50. Our results showed that the expected score for participants that have positive affect at level of −1.96 (Theta) and below, is 15.50 or less. That is, these participants are most likely to choose the answer coded 1 or 2 on most items. With critical values (−1.96 and 1.96) coding to the standard normal distribution we can expect 95% of randomly selected participants have a score between 15.50 and 46.50 (see Figure 3C).

#### Negative Affect

**FIGURE 4:**
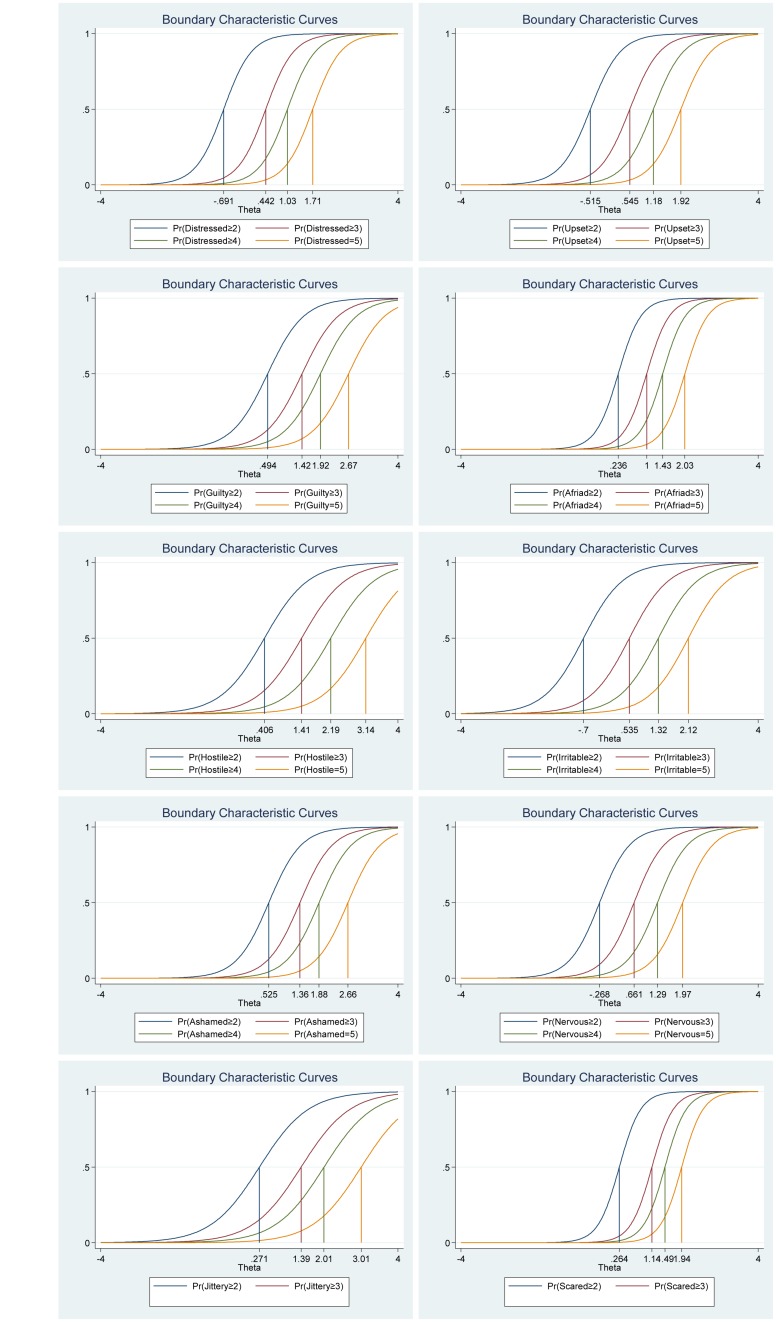
Boundary characteristic curves for each item of the negative affect scale of the Positive Affect Negative Affect Schedule (*N* = 1000).

We found that the frequency distributions for each of the items in the negative affect scale varied (see Table 4). For example, for the item “Distressed,” 7.20% of participants report a high negative affect (5 = *extremely*) compared with the item “Hostile” for which only 1.60% of participants report high negative affect (5 = *extremely*). In other words, the item “Hostile” differ in its difficulty compared with the item “Distressed” that has less difficulty (for more details see Table 5). Regarding item discrimination, all items had high discrimination values (Alphas from 1.53 to 3.49) and had a steeper slope (see Table 5 and Figure 4). Thus, indicating that that the items can differentiate well between persons with high and low levels of the latent score of negative affect. The difficulty parameters estimations (Beta) for the negative affect scale are between −0.70 and 3.14 (see Table 5). The item “Hostile” has the highest estimated difficulty parameter on response 5 (β = 3.14) and the item “Irritable” has the lowest estimated difficulty parameter on response 1 (β = −0.70). Our results also showed that the differences between categories around difficulty parameters are not equal across the negative affect scale items. For example, 5 (*extremely*) was 3.14 for the item “Hostile,” while it was 1.71 for the item “Distressed.” Moreover, the differences in difficulty varied within each item (i.e., distances between responses for each item). For example, for the item “Distressed,” the difference between ≥ 2 and ≥3 is −0.69 – (0.44) = −0.15, while the difference between ≥3 and ≥4 is 0.44 – (1.03) = 0.59. Thus, participants’ total score of negative affect will differ from totals scores using CTT, where differences are treated as equal and added without further justification (for more details see Table 5 and Figure 4).

**TABLE 4:** The frequency distributions of the negative affect scale of the Positive Affect Negative Affect Schedule (*N* = 1000).

Item	Points of Likert scale				
	1	2	3	4	5
**Distressed**					
Frequency	275	365	169	119	72
Percent	27.50	36.50	16.90	11.90	7.20
Cumulating	27.50	64.00	80.90	92.80	100.00
**Upset**					
Frequency	328	338	169	110	55
Percent	32.80	33.80	16.90	11.00	5.50
Cumulating	32.80	66.60	83.50	94.50	100.00
**Guilty**					
Frequency	647	222	64	46	21
Percent	64.70	22.20	6.40	4.60	2.10
Cumulating	64.70	86.90	93.30	97.90	100.00
**Afraid**					
Frequency	574	244	84	64	34
Percent	57.40	24.40	8.40	6.40	3.40
Cumulating	57.40	81.80	90.20	96.60	100.00
**Hostile**					
Frequency	611	230	97	46	16
Percent	61.10	23.00	9.70	4.60	1.60
Cumulating	61.10	84.10	93.80	98.40	100.00
**Irritable**					
Frequency	297	353	187	106	57
Percent	29.70	35.30	18.70	10.60	5.70
Cumulating	29.70	65.00	83.70	94.30	100.00
**Ashamed**					
Frequency	661	205	69	47	18
Percent	66.10	20.50	6.90	4.70	1.80
Cumulating	66.10	86.60	93.50	98.20	100.00
**Nervous**					
Frequency	405	301	150	92	52
Percent	40.50	30.10	15.00	9.20	5.20
Cumulating	40.50	70.60	85.60	94.80	100.00
**Jittery**					
Frequency	573	257	81	63	26
Percent	57.30	25.70	8.10	6.30	2.60
Cumulating	57.30	83.00	91.10	97.40	100.00
**Scared**					
Frequency	585	264	63	51	37
Percent	58.50	26.40	6.30	5.10	3.70
Cumulating	58.50	84.90	91.20	96.30	100.00

**TABLE 5:** Item response analysis of the negative affect scale in the Positive Affect Negative Affect Schedule (*N* = 1000).

	Coef.	*SE*	*Z*	*P*	95% CI	
**Distressed**						
Discrimination	2.66	0.15	17.57	0.00	2.36	2.96
Difficulty						
≥2	−0.69	0.05	−12.71	0.00	−0.80	−0.58
≥3	0.44	0.05	9.37	0.00	0.35	0.53
≥4	1.03	0.06	17.62	0.00	0.91	1.14
5.00	1.71	0.08	20.44	0.00	1.54	1.87
**Upset**						
Discrimination	2.47	0.14	17.37	0.00	2.19	2.75
Difficulty						
≥2	−0.52	0.05	−9.77	0.00	−0.62	−0.41
≥3	0.55	0.05	10.99	0.00	0.45	0.64
≥4	1.18	0.06	18.43	0.00	1.06	1.31
5.00	1.92	0.10	19.87	0.00	1.73	2.11
**Guilty**						
Discrimination	2.05	0.14	14.57	0.00	1.78	2.33
Difficulty						
≥2	0.49	0.05	9.35	0.00	0.39	0.60
≥3	1.42	0.08	17.34	0.00	1.26	1.58
≥4	1.92	0.11	17.75	0.00	1.70	2.13
5.00	2.67	0.17	16.15	0.00	2.35	3.00
**Afraid**						
Discrimination	3.28	0.22	14.84	0.00	2.85	3.71
Difficulty						
≥2	0.24	0.04	5.44	0.00	0.15	0.32
≥3	1.00	0.05	18.38	0.00	0.89	1.11
≥4	1.43	0.07	20.99	0.00	1.30	1.57
5.00	2.03	0.10	20.72	0.00	1.84	2.22
**Hostile**						
Discrimination	1.70	0.12	14.34	0.00	1.46	1.93
Difficulty						
≥2	0.41	0.06	7.15	0.00	0.29	0.52
≥3	1.41	0.09	15.88	0.00	1.23	1.58
≥4	2.19	0.13	16.36	0.00	1.93	2.45
5.00	3.14	0.22	14.23	0.00	2.70	3.57
**Irritable**						
Discrimination	1.89	0.11	16.95	0.00	1.67	2.11
Difficulty						
≥2	−0.70	0.06	−11.23	0.00	−0.82	−0.58
≥3	0.53	0.06	9.67	0.00	0.43	0.64
≥4	1.32	0.08	17.05	0.00	1.16	1.47
5.00	2.12	0.12	17.94	0.00	1.89	2.35
**Ashamed**						
Discrimination	2.29	0.16	14.77	0.00	1.99	2.60
Difficulty						
≥2	0.52	0.05	10.32	0.00	0.43	0.62
≥3	1.36	0.07	18.27	0.00	1.22	1.51
≥4	1.88	0.10	18.89	0.00	1.68	2.07
5.00	2.66	0.16	16.78	0.00	2.35	2.97
**Nervous**						
Discrimination	2.47	0.15	17.01	0.00	2.19	2.76
Difficulty						
≥2	−0.27	0.05	−5.41	0.00	−0.36	−0.17
≥3	0.66	0.05	12.80	0.00	0.56	0.76
≥4	1.29	0.07	18.98	0.00	1.16	1.42
5.00	1.97	0.10	19.86	0.00	1.77	2.16
**Jittery**						
Discrimination	1.53	0.11	14.21	0.00	1.32	1.74
Difficulty						
≥2	0.27	0.06	4.63	0.00	0.16	0.39
≥3	1.39	0.09	14.89	0.00	1.21	1.58
≥4	2.01	0.13	15.75	0.00	1.76	2.26
5.00	3.01	0.21	14.64	0.00	2.61	3.42
**Scared**						
Discrimination	3.49	0.24	14.34	0.00	3.01	3.97
Difficulty						
≥2	0.26	0.04	6.15	0.00	0.18	0.35
≥3	1.14	0.06	19.95	0.00	1.03	1.25
≥4	1.49	0.07	21.72	0.00	1.36	1.63
5.00	1.94	0.09	21.06	0.00	1.76	2.12

**FIGURE 5:**
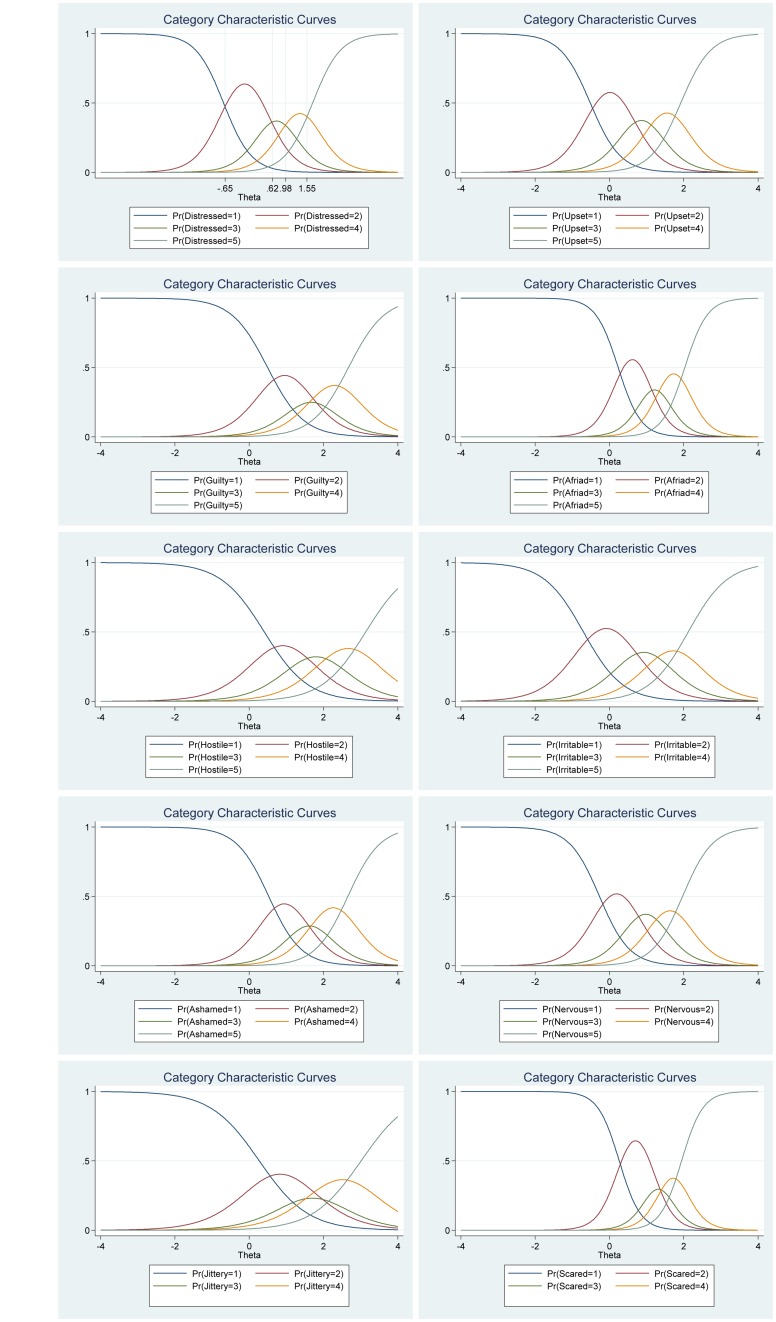
Category characteristic curves for the items in the negative affect scale of the Positive Affect Negative Affect Schedule (*N* = 1000).

Figure 5, the category characteristic curves, shows the transitions from one category to the next. For example, for the item “Distressed,” participants with negative affect (i.e., latent trait) levels below −0.65 are most likely to respond 1 (*very slightly or not at all*), while the participants with negative affect levels between 0.62 and 0.98 are most likely to respond 2, and so on. Moreover, the probability of responding 1 and 5 for this item are equal and very high (see Figure 5 for more details).

**FIGURE 6:**
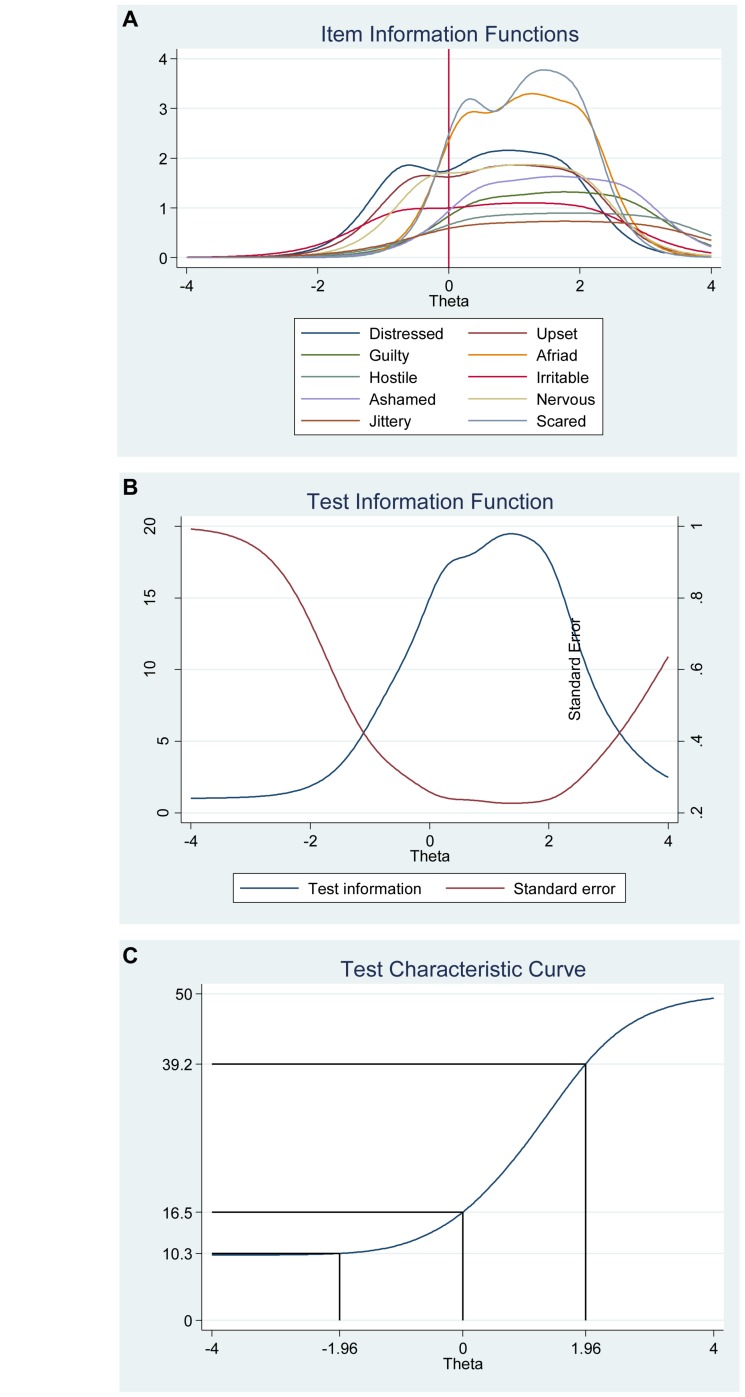
Items information function graphs for graded response with vertical line at θ = 0 **(A)** and information and standard error graph for graded response **(B)** and test characteristic curve **(C)** for the whole negative affect scale of the Positive Affect Negative Affect Schedule (*N* = 1000).

The item information function analyses indicate that the items “Scared” and “Afraid” have the two highest discrimination estimates and provide more information than the remaining items, while the items “Jittery” and “Hostile” provided the lesser information (see Figure 6A). Moreover, we show that we get reliable information at θ = 0 (vertical red line in Figure 6A) at about 2.60 from the item “Scared,” at about 1.80 from the item “Afraid,” at about 1.75 from the item “Distressed,” at about 1.70 from the items “Nervous” and “Irritable,” and so on. Moreover, the ten items together provide a lot of information to measure negative affect among participants that vary within range −1.00 up to about 3.00 (Theta) of the level of the scale of negative affect (see Figure 6B, test information function and the standard error, that is, measurement error). This means that the negative affect scale of the Positive Affect Negative Affect Schedule has good reliability and small standard error in this range. The test information highest level is located at 1.80 (Theta), thus indicating that this score has the smallest standard error and provides the most information of the negative affect scale. However, there is almost no reliable information about below −2.00 and about above 4.00 (Theta) and the standard error increases quickly for both smaller and larger Theta values. The reliability for different levels of negative affect are shown in Table 3. These results showed that the scale’s reliability is very strong at θ = −1.00, θ = 0.00, θ = 1.00, θ = 2.00, and θ = 3.00 (between 0.84 to 0.95), but that reliability is weak (0.46) at θ = −2.00 and very week (0.10) at θ = −3.00.

Figure 6C shows the test characteristic curve for the whole scale, which indicates the expected score against the latent trait of negative affect as a sum of the probabilities. Since the negative affect scale of the Positive Affect Negative Affect Schedule has 10 items with a five-point Likert scale (1 = *very slightly or not at all*, 5 = e*xtremely*), the expected scores are between 10 and 50. Our results showed that the expected score for participants that have negative affect at level of −1.96 (Theta) and below, is 10.30 or less. That is, these participants are most likely to choose the answer coded 1 on all items. With critical values (−1.96 and 1.96) coding to the standard normal distribution we can expect 95% of randomly selected participants have a score between 15.50 and 46.50 (see Figure 3C). With critical values (−1.96 and 1.96) coding to the standard normal distribution we can expect 95% of randomly selected participants have expected score between 10.30 and 39.20 (see Figure 6C).

### IRT Analyses of the Satisfaction With Life Scale

**FIGURE 7:**
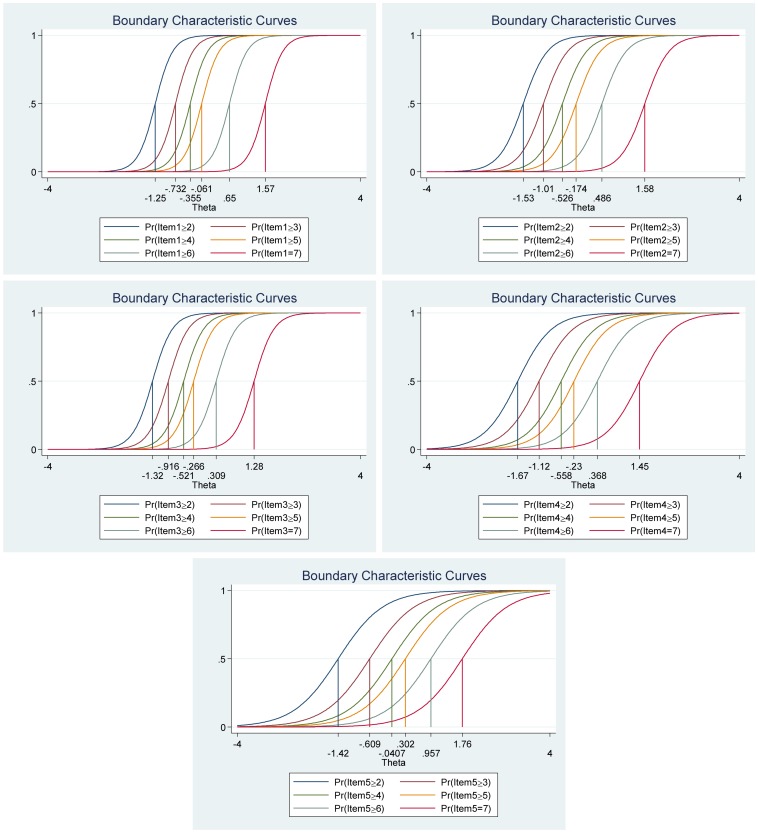
Boundary characteristic curves for each item of the Satisfaction with Life Scale (*N* = 500). Item 1: “In most ways my life is close to my ideal”; Item 2: “The conditions of my life are excellent”; Item 3: “I am satisfied with my life”; Item 4: “So far, I have gotten the important things I want in life”; and Item 5: “If I could live my life over, I would change almost nothing.”

Again, as for the positive and negative affect scales, the frequency distributions for each of the items in the Satisfaction with Life Scale varied (see Table 6). Thus, suggesting that some items differ in difficulty compared to other items in the scale. For example, for item 4 (“So far I have gotten the important things I want in life”), 12.40% of the participants reported high satisfaction with life (7 = *strongly agree*), while only 7% of the participants report 7 when answering item 1 (“In most ways my life is close to my ideal”). Moreover, all items had very high discrimination values (from 1.74 to 4.50) and a steeper slope, which indicates that the items can differentiate well between persons with high and low levels of the latent score of satisfaction with life (see Table 7 and Figure 7). In addition, the difficulty parameters estimations for the Satisfaction with Life Scale are between −1.69 and 1.76. Here, Item 5 (“If I could live my life over, I would change almost nothing”) has the highest estimated difficulty parameter on response 7 (1.76) and item 4 (“So far, I have gotten the important things I want in life”) has the lowest estimated difficulty parameter on response 1 (−1.67). Our results showed also that the differences between categories around difficulty parameters are not equal across items. This means that for item 3 (“I am satisfied with my life”), for example, a response of 7 (*strongly agree*) was 1.28, while it was 1.76 for item 5 (“If I could live my life over, I would change almost nothing”). Moreover, the differences in difficulty varied within each item (i.e., distances between responses for each item). Thus, participants’ total score of life satisfaction will differ from totals scores using CTT, where differences are treated as equal and added without further justification. For example, for item 1 (“In most ways my life is close to my ideal”), the difference between ≥2 and ≥3 is −1.25 – (−0.73) = −0.52, while the difference between ≥3 and ≥4 is −0.73 – (−0.35) = −0.38 (for more details see Table 7 and Figure 7).

**TABLE 6:** The frequency distributions of the items in the Satisfaction with Life Scale (*N* = 500).

Item	Points of Likert scale						
	1	2	3	4	5	6	7
**In most ways my life is close to my ideal**							
Frequency	61	63	61	52	128	100	35
Percent	12.20	12.60	12.20	10.40	25.60	20.00	7.00
Cumulating	12.20	24.80	37.00	47.40	73.00	93.00	100.00
**The conditions of my life are excellent**							
Frequency	45	47	68	61	115	125	39
Percent	9.00	9.40	13.60	12.20	23.00	25.00	7.80
Cumulating	9.00	18.40	32.00	44.20	67.20	92.20	100.00
**I am satisfied with my life**							
Frequency	58	42	54	43	108	137	58
Percent	11.60	8.40	10.80	8.60	21.60	27.40	11.60
Cumulating	11.60	20.00	30.80	39.40	61.00	88.40	100.00
**So far I have gotten the important things I want in life**							
Frequency	45	44	70	50	95	134	62
Percent	9.00	8.80	14.00	10.00	19.00	26.80	12.40
Cumulating	9.00	17.80	31.80	41.80	60.80	87.60	100.00
**If I could live my life over, I would change almost nothing**							
Frequency	77	85	82	50	84	70	52
Percent	15.40	17.00	16.40	10.00	16.80	14.00	10.40
Cumulating	15.40	32.40	48.80	58.80	75.60	89.60	100.00

**TABLE 7:** Item response analysis of the Satisfaction with Life Scale (*N* = 500).

	Coef.	*SE*	*Z*	*P*	95% CI	
**In most ways my life is close to my ideal**						
Discrimination	4.50	0.38	11.82	0.00	3.75	5.24
Difficulty						
≥2	–1.25	0.08	–15.38	0.00	–1.41	–1.09
≥3	–0.73	0.07	–11.21	0.00	–0.86	–0.60
≥4	–0.35	0.06	–6.00	0.00	–0.47	–0.24
≥5	–0.06	0.06	–1.07	0.29	–0.17	0.05
≥6	0.65	0.06	10.20	0.00	0.53	0.78
7	1.57	0.10	16.37	0.00	1.38	1.76
**The conditions of my life are excellent**						
Discrimination	3.25	0.24	13.66	0.00	2.78	3.72
Difficulty						
≥2	–1.53	0.10	–15.38	0.00	–1.72	–1.33
≥3	–1.01	0.08	–13.04	0.00	–1.16	–0.86
≥4	–0.53	0.07	–8.08	0.00	–0.65	–0.40
≥5	–0.17	0.06	–2.85	0.00	–0.29	–0.05
≥6	0.49	0.07	7.46	0.00	0.36	0.61
7	1.58	0.10	15.30	0.00	1.38	1.78
**I am satisfied with my life**						
Discrimination	3.93	0.31	12.70	0.00	3.33	4.54
Difficulty						
≥2	–1.32	0.09	–15.44	0.00	–1.49	–1.15
≥3	–0.92	0.07	–12.77	0.00	–1.06	–0.78
≥4	–0.52	0.06	–8.30	0.00	–0.64	–0.40
≥5	–0.27	0.06	–4.48	0.00	–0.38	–0.15
≥6	0.31	0.06	5.14	0.00	0.19	0.43
7	1.28	0.08	15.07	0.00	1.11	1.45
**So far I have gotten the important things I want in life**						
Discrimination	2.30	0.17	13.58	0.00	1.97	2.63
Difficulty						
≥2	–1.67	0.12	–14.00	0.00	–1.91	–1.44
≥3	–1.12	0.09	–12.31	0.00	–1.30	–0.94
≥4	–0.56	0.07	–7.61	0.00	–0.70	–0.41
≥5	–0.23	0.07	–3.37	0.00	–0.36	–0.10
≥6	0.37	0.07	5.25	0.00	0.23	0.51
7	1.45	0.11	13.58	0.00	1.24	1.66
**If I could live my life over, I would change almost nothing**						
Discrimination	1.74	0.14	12.79	0.00	1.47	2.01
Difficulty						
≥2	–1.42	0.12	–11.79	0.00	–1.65	–1.18
≥3	–0.61	0.09	–7.17	0.00	–0.78	–0.44
≥4	–0.04	0.08	–0.54	0.59	–0.19	0.11
≥5	0.30	0.08	3.89	0.00	0.15	0.45
≥6	0.96	0.10	9.94	0.00	0.77	1.15
7	1.76	0.14	12.50	0.00	1.48	2.04

**FIGURE 8:**
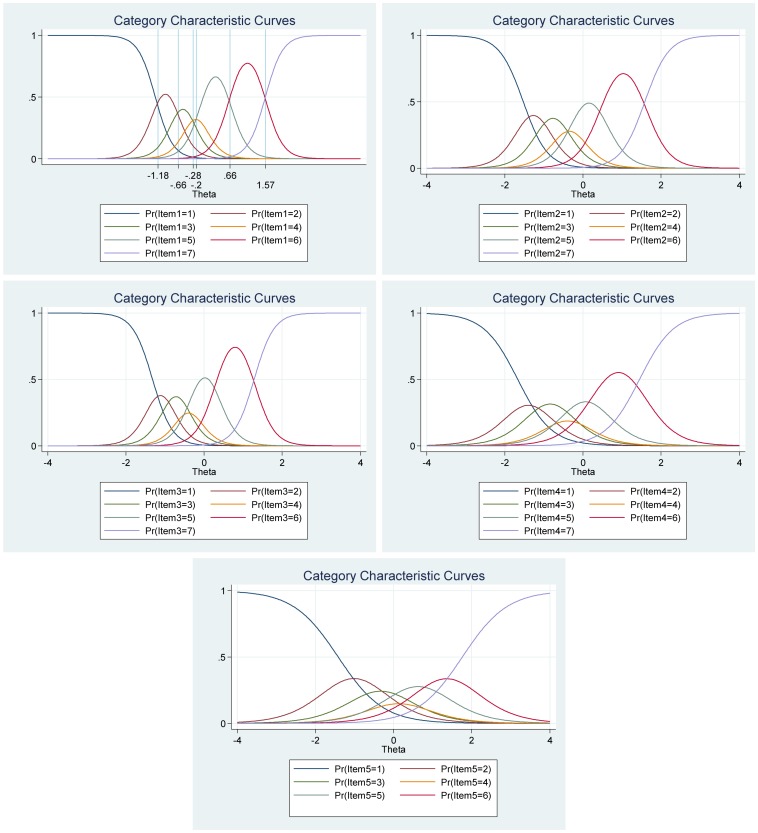
Category characteristic curves for each item of the Satisfaction with Life Scale (*N* = 500). Item 1: “In most ways my life is close to my ideal”; Item 2: “The conditions of my life are excellent”; Item 3: “I am satisfied with my life”; Item 4: “So far, I have gotten the important things I want in life”; and Item 5: “If I could live my life over, I would change almost nothing.”

Figure 8, the category characteristic curves, shows the transitions from one category to the next. For example, for item 1 (“In most ways my life is close to my ideal”), participants with satisfaction with life (latent trait) levels below -1.18 are most likely to respond 1 (*strongly disagree*), while participants with satisfaction with life levels between 1.18 and −0.66 are most likely to respond 2, and so on. Moreover, the probability of option 1 and 7 for this item are equal and very high (see Figure 8 for all the details).

**FIGURE 9:**
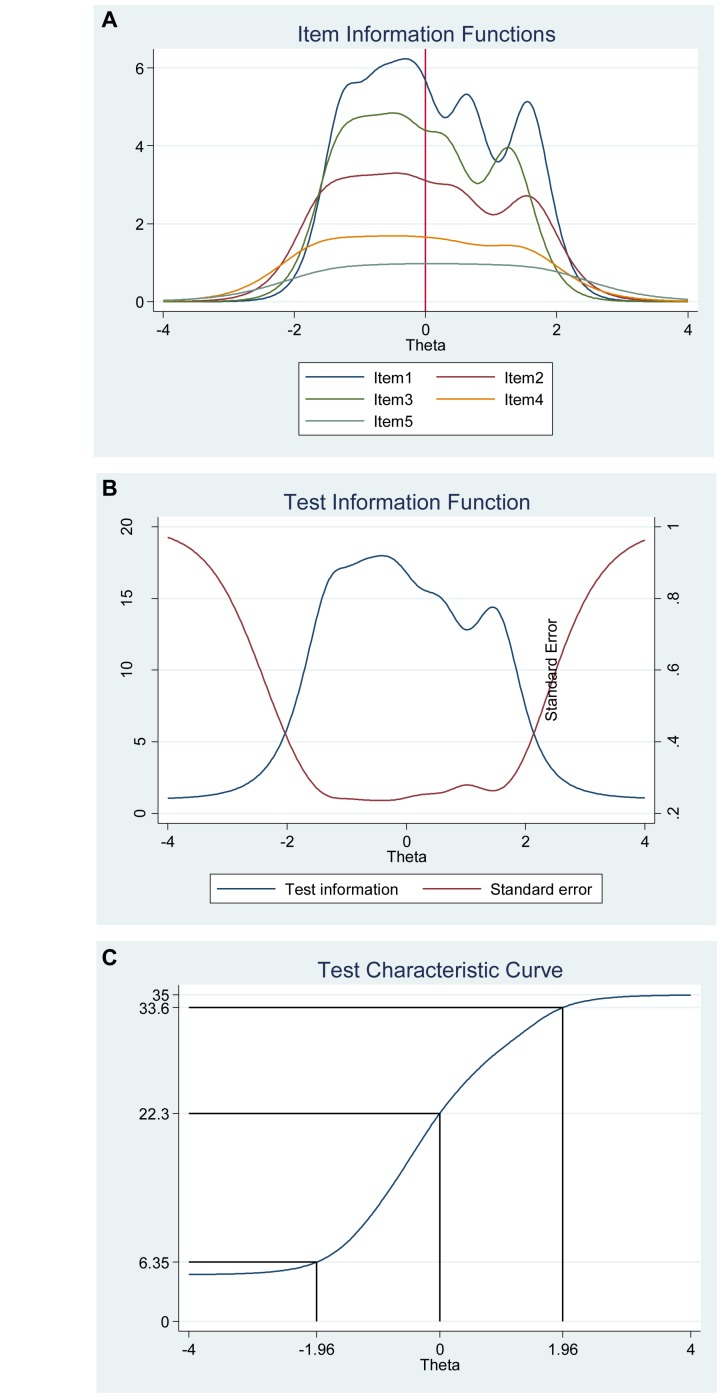
Items information function graphs for graded response with vertical line at θ = 0 **(A)** and information and standard error graph for graded response **(B)** and test characteristic curve **(C)** for the whole Satisfaction with Life Scale (*N* = 500). Note: Item 1: “In most ways my life is close to my ideal”; Item 2: “The conditions of my life are excellent”; Item 3: “I am satisfied with my life”; Item 4: “So far, I have gotten the important things I want in life”; and Item 5: “If I could live my life over, I would change almost nothing.”

The item information function analyses, Figure 9A, showed that items 1 (“In most ways my life is close to my ideal”) and item 3 (“I am satisfied with my life”) have the two highest discrimination estimates and provide more information than the remaining items, while item 5 (“If I could live my life over, I would change almost nothing”) provides lesser information. In general, the results suggest that a lot of information of the true range of life satisfaction is covered between low (Theta = −2.00) up to high (Theta = 2.00) values. Moreover, we show that we get reliable information at θ = 0.00 at about 5.80 from item 1 (“In most ways my life is close to my ideal”), at about 3.30 from item 2 (“The conditions of my life are excellent”), at about 4.30 from item 3 (“I am satisfied with my life”), at about 1.80 from item 4 (“So far, I have gotten the important things I want in life”) and at about 1.20 from item 5 (“If I could live my life over, I would change almost nothing”) (see Figure 9B, test information function and the standard error, that is, measurement error). This means that the Satisfaction with Life Scale has good reliability and small standard error in this range. The test information highest is located at about −0.30 (Theta), thus indicating that this score has the smallest standard error and provides the most information of the scale. However, there is almost no reliable information about below −2.40 and about above 2.50 (Theta) and the standard error increases quickly for both smaller and larger Theta values. The reliability for different levels of life satisfaction are shown in Table 8. These results showed that the scale’s reliability is very strong at θ = −2.00, θ = −1.00, θ = 0.00, θ = 1.00, and θ = 2.00, but that reliability is weak at θ = −3.00 and θ = 3.00. Since the Satisfaction with Life Scale has five items with a seven-point Likert scale (1 = *strongly disagree*, 7 = *strongly agree*), the expected scores are between 5 and 35. Our results showed that the expected score for participants that have life satisfaction at the level −1.96 (Theta) and below, is 6.35 or less. That is, these participants are most likely to choose the answer coded 1 on all or most items. With critical values (−1.96 and 1.96) coding to the standard normal distribution we can expect 95% of randomly selected participants to have a score between 6.35 and 33.6 (see Figure 9C).

**TABLE 8:** Reliability of the fitted graded response IRT model of the Satisfaction with Life Scale (*N* = 500) and the Harmony in Life Scale (*N* = 500).

Theta	Satisfaction with Life Scale			Harmony in Life Scale		
	Test Information Function	Test Information Function-*SE*	Reliability IRT GRM	Test Information Function	Test Information Function-*SE*	Reliability IRT GRM
−3.00	1.51	0.81	0.34	2.02	0.70	0.50
−2.00	5.93	0.41	0.83	7.94	0.35	0.87
−1.00	17.21	0.24	0.94	22.88	0.21	0.96
0.00	16.80	0.24	0.94	22.21	0.21	0.95
1.00	12.82	0.28	0.92	11.87	0.29	0.92
2.00	7.43	0.37	0.87	9.68	0.32	0.90
3.00	1.58	0.80	0.37	1.48	0.82	0.32

**Cronbach’s alpha* for the whole scale using CTT were 0.90 for the Satisfaction with Life Scale and 0.92 for the Harmony in Life Scale.*

### IRT Analyses of the Harmony in Life Scale

**FIGURE 10:**
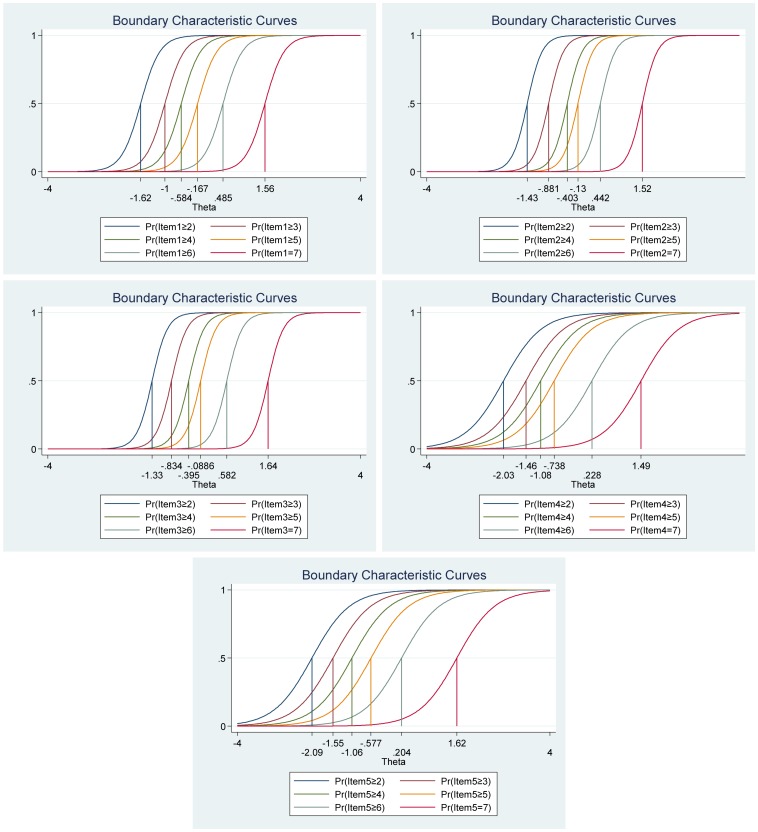
Boundary characteristic curves for each item of the Harmony in Life Scale (*N* = 500). Item 1: “My lifestyle allows me to be in harmony”; Item 2: “Most aspects of my life are in balance”; Item 3: “I am in harmony”; Item 4: “I accept the various conditions of my life”; and Item 5: “I fit in well with my surroundings.”

As for the other subjective well-being measures, the frequency distributions for each of the items in the Harmony in Life Scale varied (see Table 9). Hence, suggesting that some items differ in difficulty compared to other items in the scale. For example, while 12.20% of the participants reported harmony in life (7 = *strongly agree*) for item 4 (“I accept the various conditions of my life”), only 5.20% of the participants reported high harmony in life (7 = *strongly agree*) for item 3 (“I am in harmony”). Moreover, all items had very high discrimination values (from 2.05 to 5.23) and a steeper slope, which indicates that the items can differentiate well between persons with high and low levels of the latent score of harmony in life (see Table 10 and Figure 10). Furthermore, the difficulty parameters estimations for the Harmony in Life scale are between −2.09 and 1.64. Here, Item 3 (“I am in harmony”) has the highest estimated difficulty parameter on response 7 (1.64) and item 5 (“I fit in well with my surroundings”) has the lowest estimated difficulty parameter on response 1 (−2.09). Our result also showed that the differences between categories around difficulty parameters are not equal across items. This means that for item 3 (“I am in harmony”), for example, a response of 7 (*strongly agree*) was 1.64, while it was 1.49 for item 4 (“I accept the various conditions of my life”). Moreover, the differences in difficulty varied within each item (i.e., distances between responses for each item). Thus, participants’ total score of harmony in life will differ from totals scores using CTT, where differences are treated as equal and added without further justification. For example, for item 1 (“Most aspects of my life are in balance”), the difference between ≥2 and ≥3 is −1.62 – (−1.00) = −0.62, while the difference between ≥3 and ≥4 is −1.00− (−0.58) = −0.42 (see Table 10 and Figure 7).

**TABLE 9:** The frequency distributions of the items in the Harmony in Life Scale (*N* = 500).

Item	Points of Likert scale						
	1	2	3	4	5	6	7
**My lifestyle allows me to be in harmony**							
Frequency	35	54	55	71	120	131	34
Percent	7.00	10.80	11.00	14.20	24.00	26.20	6.80
Cumulating	7.00	17.80	28.80	43.00	67.00	93.20	100.00
**Most aspects of my life are in balance**							
Frequency	44	56	71	46	109	142	32
Percent	8.80	11.20	14.20	9.20	21.80	28.40	6.40
Cumulating	8.80	20.00	34.20	43.40	65.20	93.60	100.00
**I am in harmony**							
Frequency	53	58	64	55	126	118	26
Percent	10.60	11.60	12.80	11.00	25.20	23.60	5.20
Cumulating	10.60	22.20	35.00	46.00	71.20	94.80	100.00
**I accept the various conditions of my life**							
Frequency	32	32	33	40	145	157	61
Percent	6.40	6.40	6.60	8.00	29.00	31.40	12.20
Cumulating	6.40	12.80	19.40	27.40	56.40	87.80	100.00
**I fit in well with my surroundings**							
Frequency	28	27	44	63	118	168	52
Percent	5.60	5.40	8.80	12.60	23.60	33.60	10.40
Cumulating	5.60	11.00	19.80	32.40	56.00	89.60	100.00

**TABLE 10:** Item response analysis of the Harmony in Life Scale (*N* = 500).

	Coef.	*SE*	*Z*	*P*	95% CI	
**My lifestyle allows me to be in harmony**						
Discrimination	4.05	0.30	13.58	0.00	3.47	4.64
Difficulty						
≥2	–1.62	0.10	–16.40	0.00	–1.82	–1.43
≥3	–1.00	0.07	–13.77	0.00	–1.15	–0.86
≥4	–0.58	0.06	–9.26	0.00	–0.71	–0.46
≥5	–0.17	0.06	–2.88	0.00	–0.28	–0.05
≥6	0.48	0.06	7.88	0.00	0.36	0.61
7	1.56	0.10	15.80	0.00	1.36	1.75
**Most aspects of my life are in balance**						
Discrimination	5.23	0.44	11.82	0.00	4.37	6.10
Difficulty						
≥2	–1.43	0.09	–16.70	0.00	–1.59	–1.26
≥3	–0.88	0.07	–13.23	0.00	–1.01	–0.75
≥4	–0.40	0.06	–6.94	0.00	–0.52	–0.29
≥5	–0.13	0.06	–2.31	0.02	–0.24	–0.02
≥6	0.44	0.06	7.59	0.00	0.33	0.56
7	1.52	0.09	16.31	0.00	1.34	1.70
**I am in harmony**						
Discrimination	5.08	0.42	12.05	0.00	4.25	5.91
Difficulty						
≥2	–1.33	0.08	–16.33	0.00	–1.49	–1.17
≥3	–0.83	0.07	–12.70	0.00	–0.96	–0.71
≥4	–0.40	0.06	–6.80	0.00	–0.51	–0.28
≥5	–0.09	0.06	–1.59	0.11	–0.20	0.02
≥6	0.58	0.06	9.58	0.00	0.46	0.70
7	1.64	0.10	16.15	0.00	1.44	1.84
**I accept the various conditions of my life**						
Discrimination	2.05	0.15	13.48	0.00	1.76	2.35
Difficulty						
≥2	–2.03	0.15	–13.86	0.00	–2.32	–1.75
≥3	–1.46	0.11	–13.10	0.00	–1.68	–1.24
≥4	–1.08	0.09	–11.53	0.00	–1.27	–0.90
≥5	–0.74	0.08	–9.04	0.00	–0.90	–0.58
≥6	0.23	0.07	3.20	0.00	0.09	0.37
7	1.49	0.12	12.88	0.00	1.26	1.71
**I fit in well with my surroundings**						
Discrimination	2.06	0.15	13.59	0.00	1.76	2.36
Difficulty						
≥2	–2.09	0.15	–13.59	0.00	–2.39	–1.79
≥3	–1.55	0.12	–13.20	0.00	–1.78	–1.32
≥4	–1.06	0.09	–11.53	0.00	–1.25	–0.88
≥5	–0.58	0.08	–7.55	0.00	–0.73	–0.43
≥6	0.20	0.07	2.86	0.00	0.06	0.34
7	1.62	0.12	13.29	0.00	1.38	1.86

**FIGURE 11:**
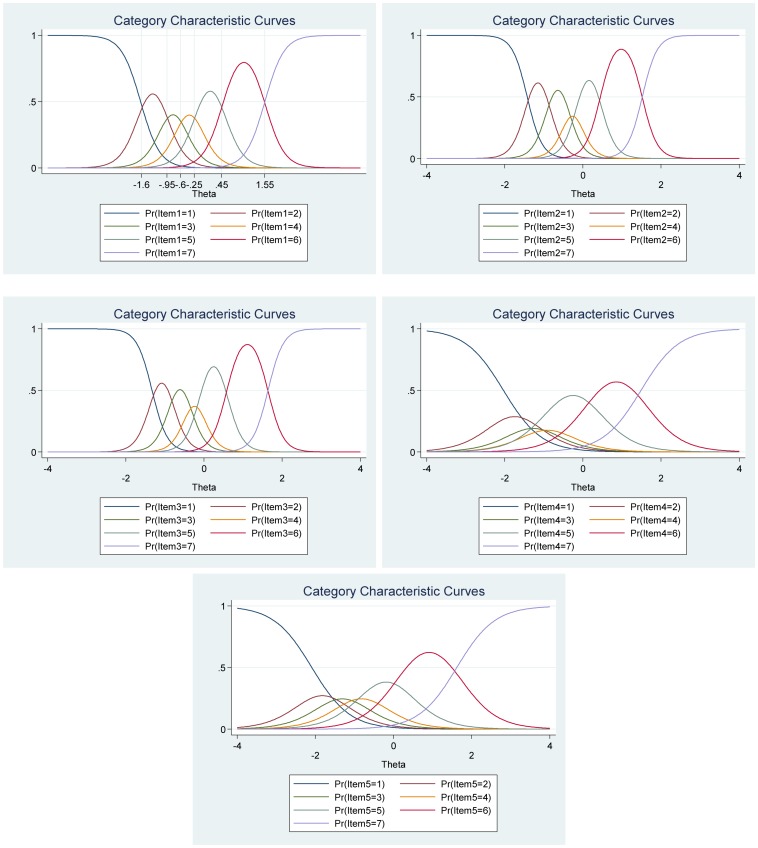
Category characteristic curves for each item of the Harmony in Life Scale (*N* = 500). Item 1: “My lifestyle allows me to be in harmony”; Item 2: “Most aspects of my life are in balance”; Item 3: “I am in harmony”; Item 4: “I accept the various conditions of my life”; and Item 5: “I fit in well with my surroundings.”

The analyses of the category characteristic curves showed that, for example, for item 1 (“My lifestyle allows me to be in harmony”), participants with harmony in life (latent trait) levels below −1.60 are most likely to respond 1 (*strongly disagree*), while participants with harmony in life levels between −1.60 and −0.95 are most likely to respond 2, and so on. Moreover, the probability of option 1 and 7 for this specific item are equal and very high (see Figure 11 for more details).

**FIGURE 12:**
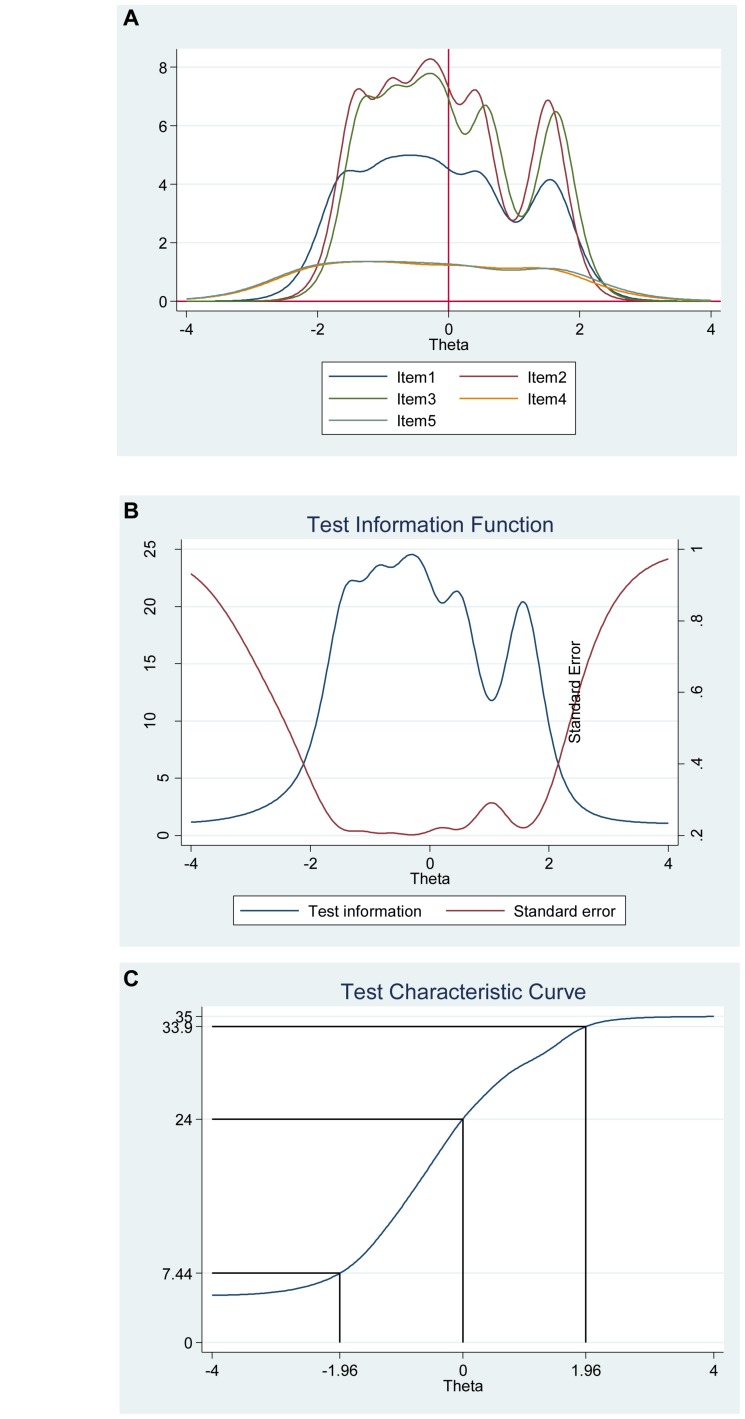
Items information function graphs for graded response with vertical line at θ = 0 **(A)** and information and standard error graph for graded response **(B)** and test characteristic curve **(C)** for the whole Satisfaction with Life Scale (*N* = 500). Item 1: “My lifestyle allows me to be in harmony”; Item 2: “Most aspects of my life are in balance”; Item 3: “I am in harmony”; Item 4: “I accept the various conditions of my life”; and Item 5: “I fit in well with my surroundings.”

The item information function analyses, Figure 12A, showed that items 2 (“Most aspects of my life are in balance”) and item 3 (“I am in harmony”) have the two highest discrimination estimates and provide more information than the remaining items, while items 4 (“I accept the various conditions of my life”) and 5 (“I fit in well with my surroundings”) provide lesser information. In general, the results suggest that a lot of information of the true range of harmony in life is covered between low (θ = −2.00) up to high (θ = 2.00) values. For instance, we showed that we get reliable information at θ = 0.00 at about 7.20 from item 2 (“Most aspects of my life are in balance”), at about 7.00 from item 3 (“I am in harmony”), at about 4.80 from item 1 (“My lifestyle allows me to be in harmony”) and at about 1.50 from both item 4 (“I accept the various conditions of my life”) and 5 (“I fit in well with my surroundings”) (see Figure 12B, test information function and the standard error, that is, measurement error). This means that the Harmony in Life Scale has good reliability and small standard error in this range. The test information highest is located at about −0.30 (Theta), hence indicating that this score has the smallest standard error and it provides the most information of the scale. However, there is almost no reliable information about below −2.40 and about above 2.50 (Theta) and the standard error increases quickly for both smaller and larger Theta values. The reliability for different levels of harmony in life are shown in Table 8. These results showed that the scales reliability is very strong at θ = −2.00, θ = −1.00, θ = 0.00, θ = 1.00, and θ = 2.00 (between 0.87 and 0.96), but weak (0.50) at θ = −3.00 and very week (0.32) at θ = 3.00.

The Harmony in Life Scale has five items with a seven-point Likert scale (1 = *strongly disagree*, 7 = *strongly agree*), so the expected scores range from 5 to 35. Our results showed that the expected score for participants that have harmony in life at the level −1.96 (Theta) and below is 7.44 and less. Hence, these participants are most likely to choose the answer coded 1 on most items. With critical values (−1.96 and 1.96) coding to the standard normal distribution, we can expect 95% of randomly selected participants have a score between 7.44 and 33.9 (see Figure 12).

### Convergent and Discriminant Validity

Finally, in order to test *convergent* and *discriminant validity* we investigated the Pearson correlations between the different scales. The Satisfaction with Life Scale (*r* = 0.30; *p* < 0.001) and Harmony in Life Scale (*r* = 0.46; *p* < 0.001) were positively and significantly correlated with the positive affect scale. Conversely, the Satisfaction with Life Scale (*r* = −0.30; *p* < 0.001) and Harmony in Life Scale (*r* = −0.38; *p* < 0.001) were negatively and significantly correlated with the negative affect scale. Moreover, positive and negative were negatively and significantly correlated with each other (*r* = −0.25; *p* < 0.001). Hence, there is sufficient convergent and discriminant validity.

## Discussion

Since measures used to assess subjective well-being are self-reports, often validated only using CTT methodology, our aim was to focus on the psychometric properties of three subjective well-being measures using IRT methods. More specifically, we used GRM to validate and suggest psychometric modifications to the Positive Affect Negative Affect Schedule, the Satisfaction with Life, and the Harmony in Life Scale. We argued that health is biopsychosocial and suggested that these three scales operationalize a biopsychosocial model of subjective well-being (cf. affect-cognition-behavior). Since past research shows that each scale has a unidimensional structure, our first step here was to validate each scale at the item level.

### The Affective or Biological Component: Positive Affect Negative Affect Schedule

The results showed that, despite having a varied frequency distribution, all items measuring positive and negative affect had high discrimination values (Alphas from 1.37 to 2.65 for positive affect and 1.53 to 3.49 for negative affect). In other words, indicating that all items in the scales can differentiate well between persons with high and low levels of positive and negative affect. Moreover, certain items had different difficulty parameter (Beta) for each specific response option. For example, participants were relatively less prone to choose the highest point in the Likert scale (5 = *Extremely*) when evaluating to which extent they have felt *alert* and *hostile* and more prone to choose this response when evaluating to which extent they have felt *determined* and *distressed*. In addition, participants were relatively more prone to choose the lowest point in the Likert scale (1 = *Very slightly or not at all*) when evaluating to which extent they have felt *proud* and *ashamed* and less prone to choose this response when evaluating to which extent they have felt *interested* and *irritable*. In this context, validation studies using CTT (e.g., Crawford and Henry, 2004) suggest that best-fitting models are achieved by specifying correlations between error in items closely related to each other in meaning, for example, Interested-Alert-Attentive, Proud-Determined, Excited-Enthusiastic-Inspired, Distressed-Upset, Guilty-Ashamed, Scared-Afraid, Nervous-Jittery, Hostile-Irritable. Therefore, researchers have suggested that these covariances, that form constellations of items, indicate the possibility of item reduction without serious repercussions on the content domain or internal consistency reliability of the scales (e.g., Thompson, 2007, 2017). For instance, the CFA analysis conducted in our study to replicate the unidimensionality of the scales showed similar covariance between errors regarding Alert-Attentive and even more for the negative affect scale. Nevertheless, our IRT results suggest that choosing which item to delete is more complex than just looking at the covariances between items closely related in meaning. For instance, for the constellation Proud-Determined, “Determined” was here shown to cover the highest levels of the Likert scale and “Proud” to be able to cover the lowest levels and for the constellation Guilty-Ashamed, we need to consider that, “Guilty” covers the lowest, while “Distressed” from the constellation Distressed-Upset covers the highest levels of the Likert scale. So, deleting any of these two items has repercussions for which item should be kept from other item constellations, since the scale will need an item that covers for lower/higher values. In other words, in contrast to what is implied by CTT models, the deletion of any of these items will have repercussions on the psychometric properties of the scale.

Furthermore, the items “Enthusiastic,” “Excited,” “Proud,” “Interested,” “Strong,” “Scared,” “Afraid,” “Distressed,” “Irritable,” and “Nervous” provided satisfactory information values and seem useful to differentiate well between respondents. More specifically, the items “Enthusiastic,” “Excited,” “Scared,” and “Afraid” had two of the highest discrimination estimates (Alpha) and provided more information than all the remaining items, while the items “Alert,” “Attentive,” “Jittery,” and “Hostile” provided lesser information. Moreover, the test’s highest amount of information was located within positive affect levels from −2.50 up to about 2.30 and within negative affect levels from −1.00 up to about 3.50 (Theta). However, even if some items, like “Alert” and “Attentive,” had good discrimination values (Alpha), the information value was low. Hence, suggesting again that the item “Alert” can be removed, or even better, replaced with an equally good discriminating item that better covers lower values of the scale and provides more information for the whole ideal range (Theta −3.00 to +3.00). Last but not the least, reliability was relatively week for responses were Theta is at or above 3.00 for positive affect and at and below −2.00 for negative affect, suggesting that the standard error increases quickly for higher values of positive and negative affect. Hence, choosing deletion or addition of items that cover the ideal range of affect (Theta −3.00 to +3.00) needs to consider items that complement each other in their difficulty and discrimination levels. In general, in addition to what is implied by CTT models, the information provided in our study should be useful for further development of the scales of the Positive Affect Negative Affect Schedule.

### The Cognitive or Psychological Component: The Satisfaction With Life Scale

As for the results of the affective component measure, all items of the Satisfaction with Life Scale had a varied frequency distribution and can differentiate well between persons with high and low levels of the latent score of life satisfaction (Alphas from 1.74 to 4.50). Moreover, certain items had different difficulty parameter (Beta) for each specific response option. For example, participants were relatively less prone to choose the highest point in the Likert scale (7 = *Extremely agree*) when evaluating the statement in item 5 (“If I could live my life over, I would change almost nothing”) and more prone to choose this response when evaluating the statement in item 3 (“I am satisfied with my life”). In this context, studies using CTT methods suggest that the fifth item of the scale shows often lower factor loadings and item-total correlations than the first four items of the scale (e.g., Senécal et al., 2000; see also our CFA analysis for this scale, which replicate these results in the Supplementary Material). We agree with Pavot and Diener (2008) who suggested that, because this specific item strongly implies a summary evaluation over past years, responses to it might involve a different cognitive recollection than the responses to items that imply a focus on, for example, a temporal summation (e.g., Item 3: “I am satisfied with my life”). Moreover, as in our study, the few studies using IRT methodology indicate that the fifth item is somewhat distinct from the other four items of the scale, something that makes comparisons based on raw scores in certain populations misleading (e.g., Vittersø et al., 2005; Oishi, 2006). In addition, participants were relatively more prone to choose the lowest point in the Likert scale (1 = *Extremely disagree*) when evaluating item 1 (“In most ways my life is close to my ideal”), and less prone to choose this response when evaluating item 4 (“So far I have gotten the important things I want in life”). We interpret this as participants not seeing “get the important things in my life” as equal to being close to their own self-imposed ideal, which per definition is how life satisfaction has been conceptualized (Diener et al., 1985; Pavot and Diener, 1993, 2008). Thus, suggesting that responses to these items will have repercussions on the psychometric properties of the Satisfaction with Life Scale and to comparisons between groups based on raw scores of the scale (cf. Oishi, 2006). In this line, CTT methods suggest that a life satisfaction score of 20 represents the neutral point on the scale, while a scores between 5 and 9 indicates that the respondent is extremely dissatisfied with life, scores from 15 to 19 are interpreted as falling in the slightly dissatisfied range, scores between 21 and 25 represent slightly satisfied, and scores between 31 and 35 indicate that the respondent is extremely satisfied with life (Pavot and Diener, 2008). In contrast, our IRT analysis suggest a score of 22.30 as the neutral point of the scale and that 95% of the participants are within scores 6.35–33.60. Thus, IRT might be useful to create normative data for this scale and the others.

In general terms, however, item 1 (“In most ways my life is close to my ideal”), item 2 (“The conditions of my life are excellent”), item 3 (“I am satisfied with my life”), and item 4 (“So far I have gotten the important things I want in life”) provided satisfactory information values and could differentiate well between respondents. Specifically, item 1 and 3 have the highest discrimination estimates (Alphas) and provide more information than the remaining items. The test’s highest amount of information was located within life satisfaction levels from −2.00 up to about 2.00 (Theta). Additionally, although item 5 had very high discrimination values (Alpha), it provided low information. Hence, reinforcing that item 5 should be removed or modified to develop the psychometric properties of the scale and that there is no reliable information for Theta values at and about below −2.40 and at and about above 2.50. In these specific location coefficients, the standard error increases quickly, thus, the scale’s reliability is very weak. The information provided in our study should be useful for further development of the Satisfaction with Life Scale in order to cover the ideal range of the scale (Theta −3.00 to +3.00).

### The Behavioral or Social Component: Harmony in Life Scale

As for the results of the other subjective well-being measures, the items of the Harmony in Life Scale showed varied frequency distribution, high discrimination values (Alphas from 2.05 to 5.23) and had different difficulty parameters (Beta) on each specific response option. Here, participants were relatively less prone to choose the highest point in the Likert scale (7 = *Extremely agree*) when evaluating the statement in item 3 (“I am in harmony”) and more prone to choose this response when evaluating the statement in item 4 (“I accept the various conditions of my life”). Moreover, participants were relatively more prone to choose the lowest point in the Likert scale (1 = *Extremely disagree*) when evaluating the statement in item 3 (“I am in harmony”) and less prone to choose this response when evaluating the statement in item 5 (“I fit in well with my surroundings”). In addition, items 2 (“Most aspects of my life are in balance”) and 3 (“I am in harmony”) have the highest discrimination estimates (Alpha) and provide more information than the remaining items. These two items together with item 1 (“My lifestyle allows me to be in harmony”) provide satisfactory information values, thus, they differentiate well between respondents with high and low levels in harmony in life. Although beyond the scope of our study, we argue that these results reinforce our suggestion about seeing harmony in life as the behavioral or social component of subjective well-being. All relevant items suggest evaluations of behaviors (e.g., “My lifestyle…”) and evaluations of social interactions between the self and the world around (e.g., “…in balance”).

In addition, although item 4 (“I accept the various conditions of my life”) and 5 (“I fit in well with my surroundings”) had very high discrimination values (Alphas), the information that these items cover is low. With regard to item 4, the statement is probably more related to the concept of self-acceptance, rather than harmony *per se*. Self-acceptance has been conceptualized as one sub-trait in the personality trait of Self-directedness (Cloninger, 2004). In other words, even if self-acceptance has been identified as an important trait that promotes well-being, it is a personality trait rather than a construct of subjective well-being. With regard to item 5, perhaps the word “surroundings” is too narrow or confuses the respondents. In other words, “surroundings” might be misinterpreted only as the physical environment or adjacent area, which stands in contrast to both the concept of harmony as the sense of balance and flexibility that an individual experience in relation to the *world* around her (Li, 2008a, b) and the way people describe *how* they pursue harmony—that is, using words that describe more than just adjacent areas, such as, *nature*; in contrast to words people use to describe *how* they pursue life satisfaction, such as, *job* and *house* (see Kjell et al., 2016), which might be what some respondents interpret as their “surroundings.” A tentative modification, for example, could be to change the statement in item 5 to “I fit in well with the world around me (e.g., nature).”

Last but not the least, the test’s highest amount of information was located within Theta values from −2.00 up to about 2.00 and the scale has almost no reliable information for Theta values at and below −2.40 and at and about above 2.50. At these values, reliability is week and the standard error increases quickly. Hence, as for the other measures, our results are useful for further development of the Harmony in Life Scale in order to cover the ideal range of the scale (Theta −3.00 to +3.00).

### Strengths and Limitations of the Present Study

IRT methodology is different from CTT in several important ways (see Hambleton and Swaminathan, 1985; Embretson and Reise, 2000 for details). One of the most significant differences is that in CTT the standard error of measurement is assumed to apply to the whole sample, while in IRT it varies depending on the latent trait score. Using IRT allowed us to consider additional sources of error, such as a person’s latent score and person-by-item interaction (Oishi, 2007). In contrast, CTT indices such as *Cronbach’s Alpha* do not provide information whether some items measured some individuals’ evaluations of their subjective well-being better than others (Oishi, 2007). As showed here, the first take home message is that there was less reliability for respondents with extreme latent scores of the different components of subjective well-being. Thus, we have suggested the need of modification or addition of specific items in order to improve reliability at the level of the scale, at the item level and at the level of the response scale for each item. This, however, is complex since our results imply that we need to consider both difficulty and discrimination scores and not only covariances between items as suggested by CTT methods. Importantly, in CTT, if two respondents answered the same number of items with the highest/lowest point in the scale, they will get the same total score even if they answered different items as high/low. In contrast, in IRT, the person who answered high to the most “difficult” items (i.e., the items less frequently answered as high) would receive a higher total score than the person who answered high to less difficult items. In addition, since IRT parameters are not sample dependent as in CTT, the score computed in IRT can be compared across different test forms and samples (Oishi, 2007). Hence, the data presented here can be used as normative data for each of the subjective well-being constructs.

Nevertheless, IRT methodology does not address the issue of response style or social desirability (cf. Oishi, 2007). For instance, item difficulty parameters might be influenced by response tendencies such as a mid-point use or extreme scale use (Oishi, 2007; see Chen et al., 1995, for cultural differences in response tendencies). Also, social desirability for specific items might be different across individuals depending on their culture or personal goals and values. For instance, items that we identified as more difficult (e.g., “Proud” in the Positive Affect Negative Affect Scale; item 5, “If I could live my life over, I would change almost nothing,” in the Satisfaction with Life Scale; and item 3, “I am in harmony,” in the Harmony in Life scale) might be seen as socially undesirable to endorse at the highest point of the scales among individuals who value modesty (cf. Oishi, 2007; see Kitayama and Markus, 2000, for cross-cultural studies on happiness). Hence, since we cannot account if our IRT results have been affected by response tendencies and social desirability, our suggestions for modifications should be interpreted as guidelines rather than rules (Oishi, 2007).

Finally, the basic 1-factor CFA model used in this study showed that some fit indexes were slightly outside the traditional acceptable range. The high values of REMSEA, for example, may suggest that the high large residuals in these models could be caused by latent multidimensional structure in the data, so this did not allow us to strongly confirm the unidimensionality of our data and cast doubts concerning the remaining dimensionality. Indeed, the result regarding local independence showed that the residuals were mostly significantly correlated, thus indicating also that the data had tendency for multidimensionality. We recommend that further research should apply both *Bifactor analysis* and *multidimensional item response theory* (MIRT) to investigate any multidimensionality regarding these measures. Tentatively, this multidimensionality, we argue, is related to our assumption of a general factor for subjective well-being (i.e., the biopsychosocial model of subjective well-being).

## Conclusion and Final Remarks

In sum, all subjective well-being measures showed varied frequency distribution, high discrimination values (Alphas), and had different difficulty parameters (Beta) on each response options. For example, we identified items that respondents found difficult to endorse at the highest and lowest points of the scale. In addition, while all scales could cover a good portion of the latent trait of subjective well-being, there was less reliability for respondents with scores at the extremes of the scales. The affective component seems to be less accurately measured, especially the negative affect scale; while the measures for both the cognitive and social components seem to cover equal range of each latent construct. Although, the scales can be modified by deletion/addition of items that have less/more difficulty to cover the ideal range of subjective well-being, in contrast to what is implied by only focusing on CTT models, the deletion/addition of items needs to consider the additional sources of error we found here. We suggest the replication of our results and the use of other methods or a combination of methods before modifications are implemented. For instance, in recent studies our research team has used artificial intelligence to use words and narratives in relation to the measurement of health (Kjell et al., 2019), subjective well-being (Garcia and Sikström, 2013), happiness (Garcia et al., 2016; Garcia et al., 2020b), and personality (Garcia and Sikström, 2014, 2019; Garcia et al., 2015; Garcia et al., 2020a, c). In one study, the scales used here seem to be related to both different and similar words people use to describe what they *relate* to the concept of happiness and what *makes* them happy (Garcia et al., 2020b). These advanced and innovative techniques can probably be applied to validate items and constructs using peoples own narratives—a method we tentatively call Quantitative Semantics Test Theory, QuSTT. Together with CTT, IRT and qualitative methods, QuSTT might contribute to more rigorous systematic process for item deletion/addition (Sikström and Garcia, 2020). Indeed, many researchers have accurately pointed out the need for improvement in the conceptualization and measurement of well-being using good qualitative, intuitive and quantitative methodology, and consideration and implementation of past research (for critical positive psychology see Brown et al., 2018).

Here, we have argued (see also Garcia et al., 2020b) that these three scales operationalize a biopsychosocial model of subjective well-being (cf. affect-cognition-behavior). We only apply the logic of health being physical, mental, and social to the concept of subjective well-being (cf. World Health Organization [WHO], 1946; Engel, 1980; Cloninger, 2004). Since past research suggests that the proposed scales measuring these constructs are unidimensional, our first step was to validate each scale at the item level. Nevertheless, we need to acknowledge that a holistic view of the human being consists of body, mind and psyche, hence, also spiritual or existential components need to be adapted and tested for a more robust and accurate conceptualization of subjective well-being (Ryff, 1989; cf. Cloninger, 2004; Vaillant, 2008; VanderWeele, 2017; MacDonald, 2018). How this is done, is important because without good measurement to discern the actual concept of subjective well-being, without understanding that it is in itself a complex system (cf. Cloninger, 2004), and without considering how people express their well-being and past relevant research beyond a specific field (e.g., the biopsychosocial model of health), we risk ending up with “quick and dirty measures” that lack a comprehensive theory (cf. Wong and Roy, 2018) and suffer of “jingle-jangle” fallacy^6^ (cf. Block, 1995).

“Let no one ignorant of geometry enter”

Plato

## Data Availability Statement

The raw data supporting the conclusions of this article will be made available by the authors, without undue reservation, to any qualified researcher.

## Ethics Statement

Ethics approval was not required at the time the research was conducted as per national regulations. The consent of the participants was obtained by virtue of survey completion after they were provided with all relevant information about the research (e.g., anonymity).

## Author Contributions

AN and DG conceived, designed, and performed the experiments, analyzed the data, wrote the manuscript, prepared the figures and/or tables, and reviewed drafts of the manuscript. KC, BP, and SS reviewed drafts of the manuscript.

## Conflict of Interest

The authors declare that the research was conducted in the absence of any commercial or financial relationships that could be construed as a potential conflict of interest.

## Footnotes

1 There are different well-validated scales that can be used to measure each component of subjective well-being, for a compilation of the most common, the reader is advised to see Lopez and Snyder (2004).

2 Throughout the manuscript the term “difficult” or “difficulty” refers to “endorsement rate” or “probability of endorsement.”

3 Amazon’s Mechanical Turk MTurk allows data collectors to recruit participants (i.e., workers) online for completing different tasks for money (for a review on the validity of this method for data collection see among others: Buhrmester et al., 2011; Rand, 2012).

4 http://www.mturk.com/mturk/welcome

5 Some researchers, however, confirm that IRT analyses are reasonably robust to violations to unidimensional factor structure assumptions (Ip, 2010).

6 Jingle refers to two constructs with equivalent labels that really reflect different phenomena, whereas jangle refers to when one construct is given multiple names (Kelley, 1927; Block, 1995).
