# Supplementary material for: Validation of Subjective Well-Being Measures Using Item Response Theory
Source: Front Psychol. 2020 Jan 22;10:3036. doi: 10.3389/fpsyg.2019.03036 (PMC6987476; doi:10.3389/fpsyg.2019.03036)
Supplement: Supplementary file 1 [file Table_1.pdf]

SUPPLEMENTARY MATERIAL

Validation of Subjective Well-Being Measures using Item Response Theory

Ali Al Nima[1, 2]*, Kevin M. Cloninger[1, 3], Björn N. Persson[1, 4], Sverker Sikström[5], Danilo Garcia[1, 2, 6]*

[1]Blekinge Center of Competence, Region Blekinge, Karlskrona, Sweden

[2]Depeartment of Psychology, University of Gothenburg, Gothenburg, Sweden

[3]Anthropedia Foundation, St. Louis, Missouri, USA

[4] Department of Psychology, University of Turku, Turku, Finland

[5]Depeartment of Psychology, Lund University, Lund, Sweden

[6]Department of Behavioral Science and Learning, Linköping University, Linköping, Sweden

* Correspondence concerning this article should be addressed to A. A. Nima, alinor_1979@yahoo.co.uk, or to D. Garcia, danilo.garcia@icloud.com.

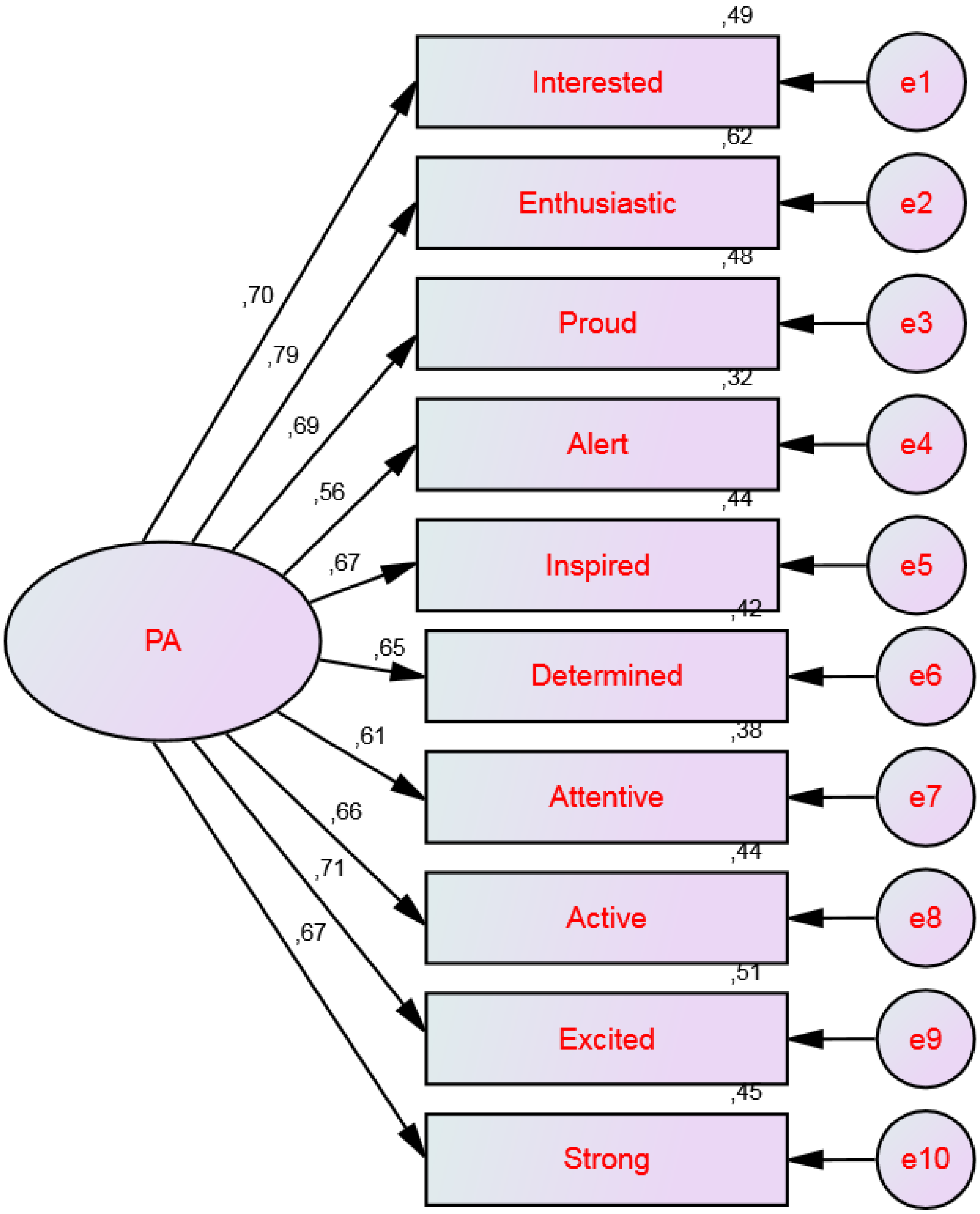

Figure S1: The basic single factor CFA model for positive affect (PA).

Note: *Chi-square* = 443.59, *df* = 35, *p* < .001; The *goodness of fit index* was .91; the *incremental fit index* was .91: and the *Root Mean Square Error of Approximation* fit statistic that was .108. All factor loadings are significant at *p* < 001.

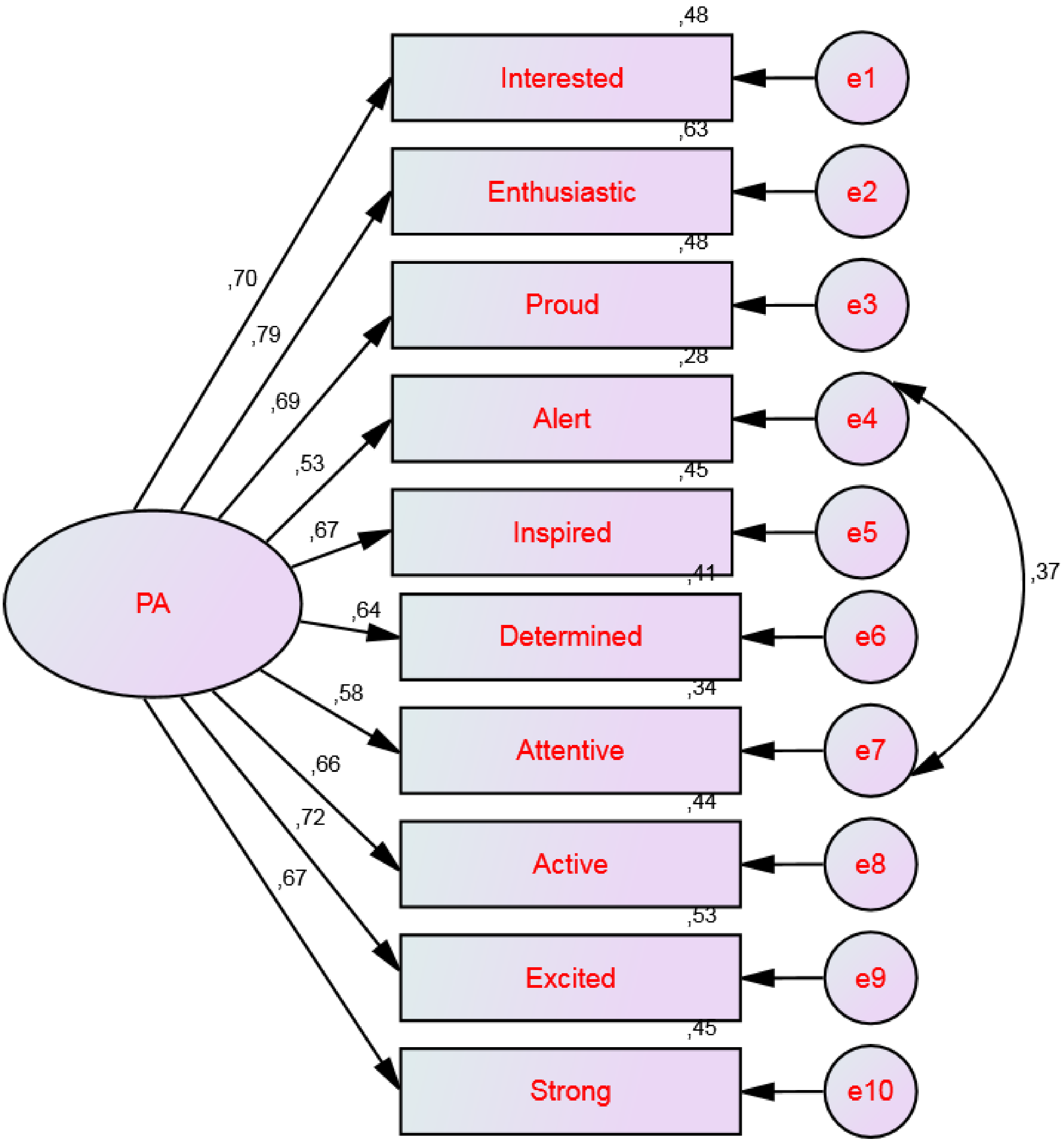

Figure S2: The basic single factor CFA model with one modification for positive affect (PA).

Note: *Chi-square* =307.55, *df* = 34, *p* < .001; The *goodness of fit index* was .94; the *incremental fit index* was .94: and the *Root Mean Square Error of Approximation* fit statistic that was .09. All factor loadings are significant at *p* < 001.

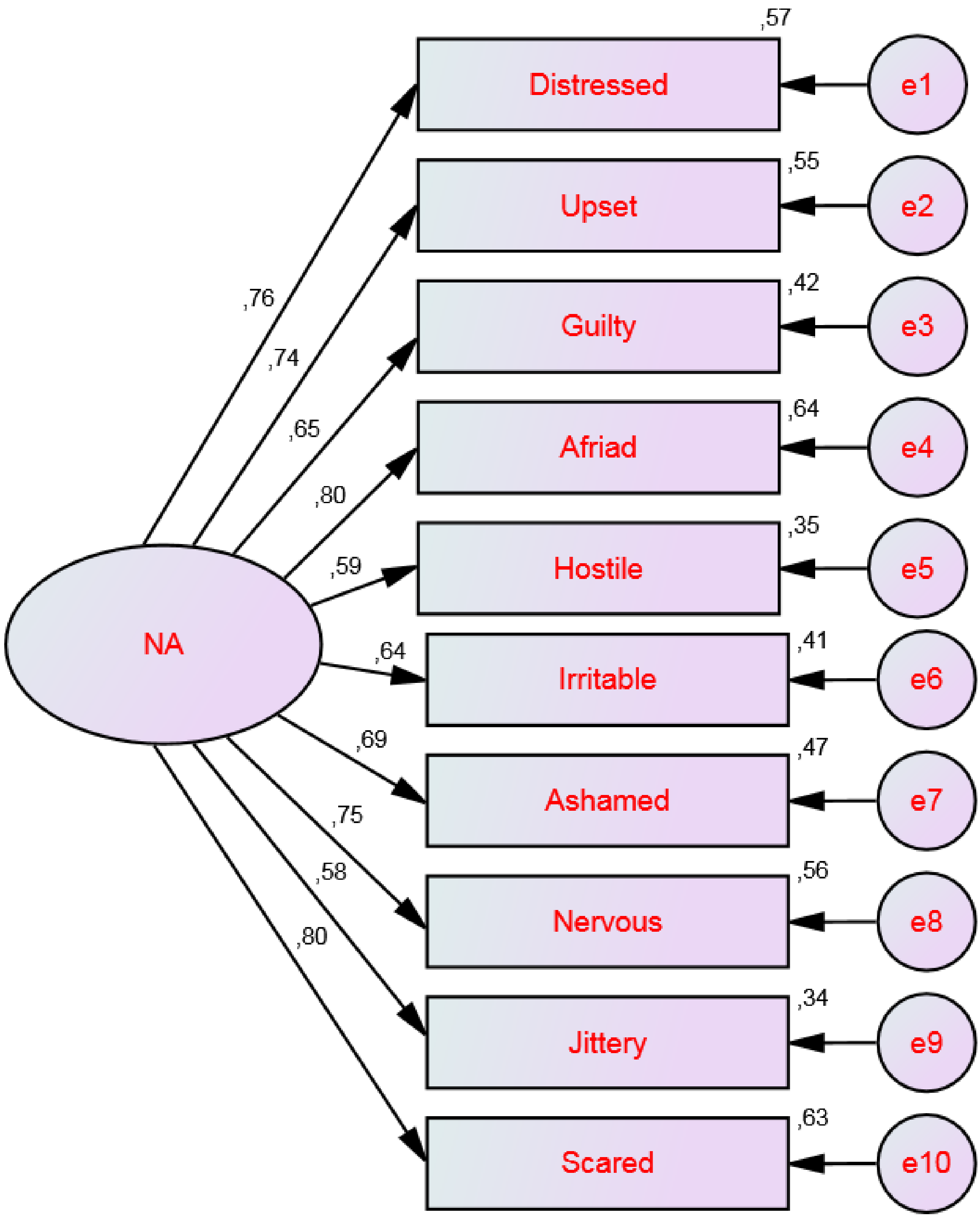

Figure S3: The basic single factor CFA model for negative affect (NA).

Note: *Chi-square* =1055.38, *df* = 35, *p* < .001; The *goodness of fit index* was .80; the *incremental fit index* was .82: and the *Root Mean Square Error of Approximation* fit statistic that was .17. All factor loadings are significant at *p* < 001.

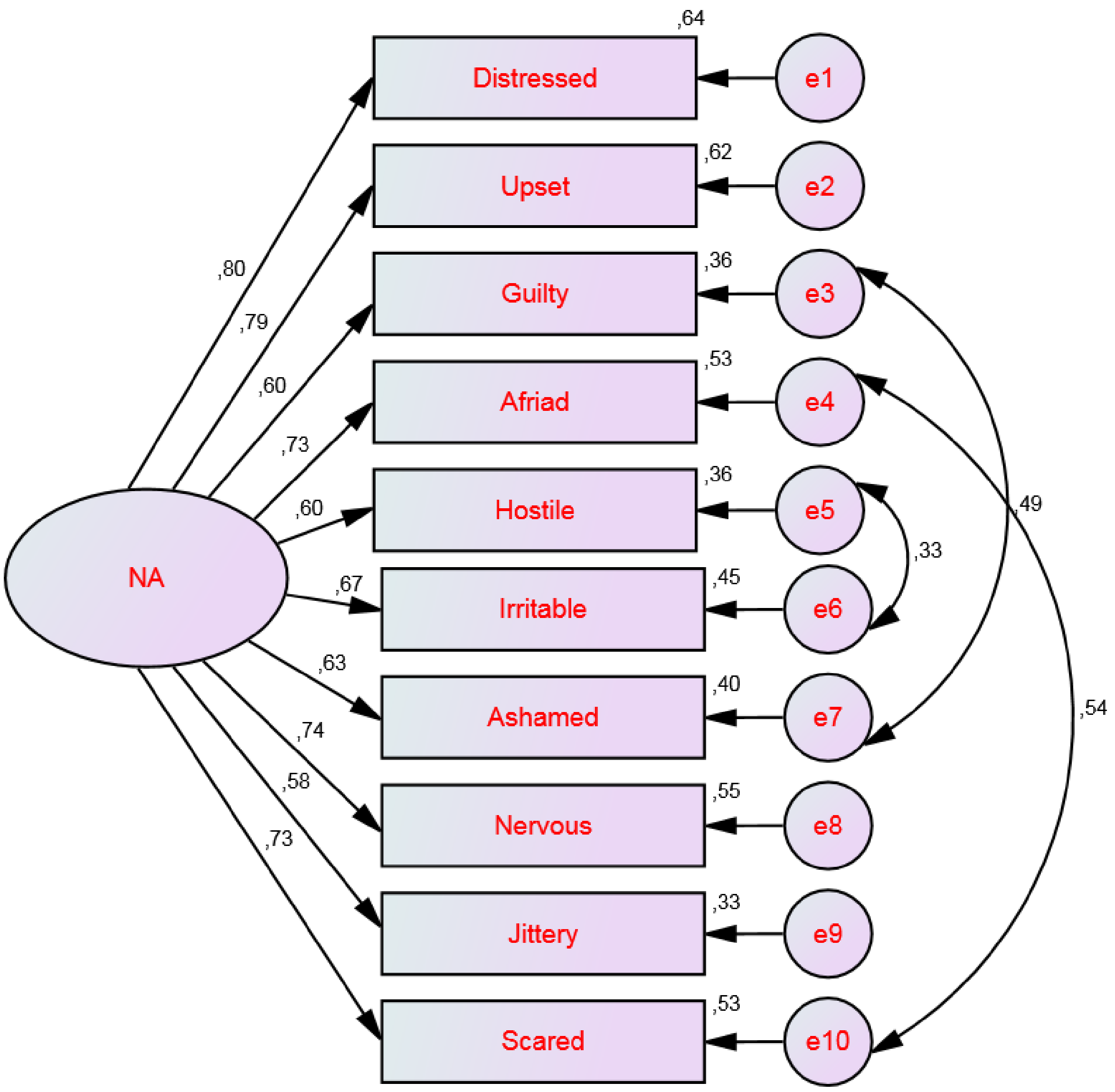

Figure S4: The basic single factor CFA model with three modifications for negative affect (NA).

Note: *Chi-square* = 438.53, *df* = 32, $p < .001$; The *goodness of fit index* was .91; the *incremental fit index* was .93: and the *Root Mean Square Error of Approximation* fit statistic that was .11. All factor loadings are significant at $p < 001$.

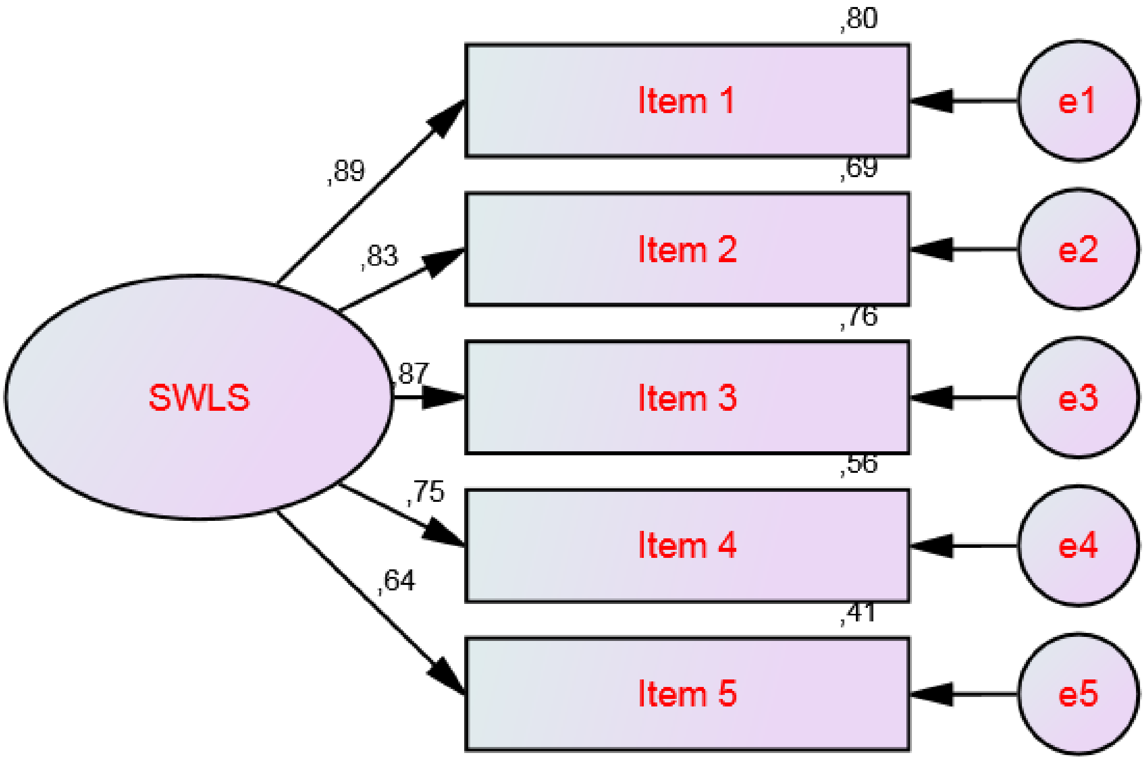

Figure S5: The basic single factor CFA model for Satisfaction with Life Scale (SWLS).

Note: *Chi-square* = 10.14, *df* = 5, *p* = .07; The *goodness of fit index* was .99; the *incremental fit index* was 1.00: and the *Root Mean Square Error of Approximation* fit statistic that was .04. All factor loadings are significant at *p* < 001. Item 1: "In most ways my life is close to my ideal"; Item 2: "The conditions of my life are excellent"; Item 3: "I am satisfied with my life"; Item 4: "So far, I have gotten the important things I want in life"; and Item 5: "If I could live my life over, I would change almost nothing".

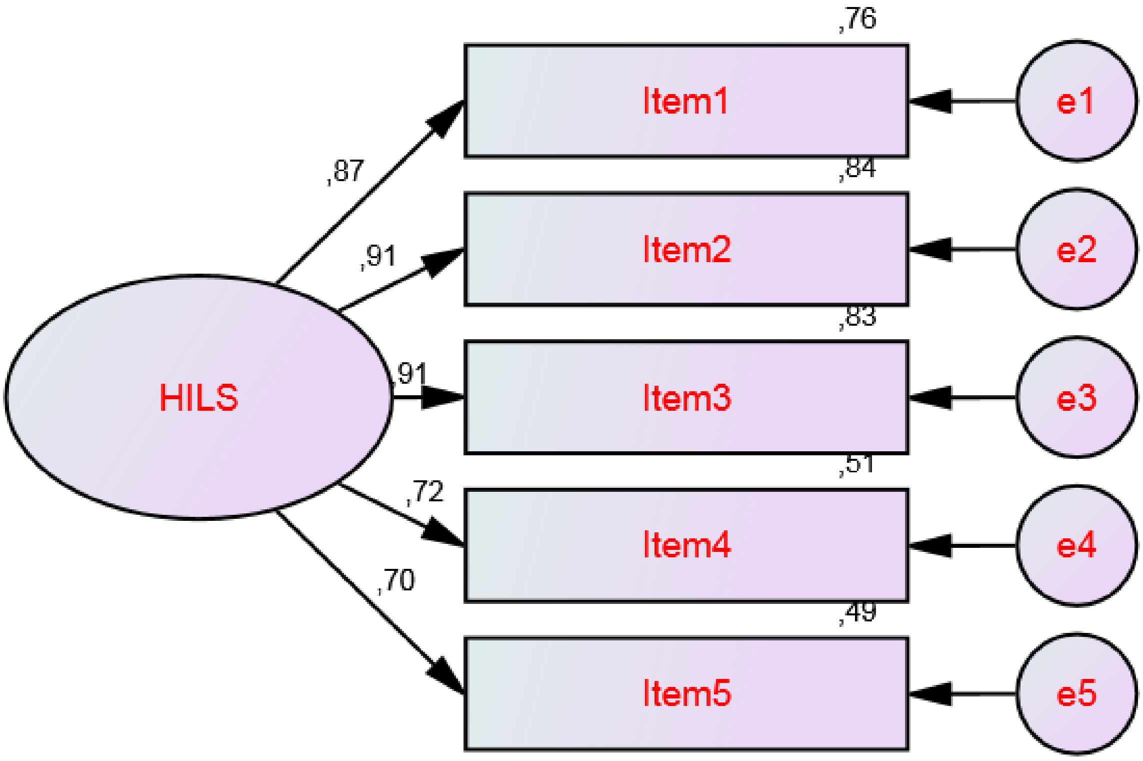

Figure S6: The basic single factor CFA model for Harmony in Life Scale (HILS).

Note: *Chi-square* =31.68, *df* = 5, *p* < .001; The *goodness of fit index* was .98; the *incremental fit index* was .99: and the *Root Mean Square Error of Approximation* fit statistic that was .10. All factor loadings are significant at *p* < 001. Item 1: “My lifestyle allows me to be in harmony”; Item 2: “Most aspects of my life are in balance”; Item 3: “I am in harmony”; Item 4: “I accept the various conditions of my life”; and Item 5: “I fit in well with my surroundings”.

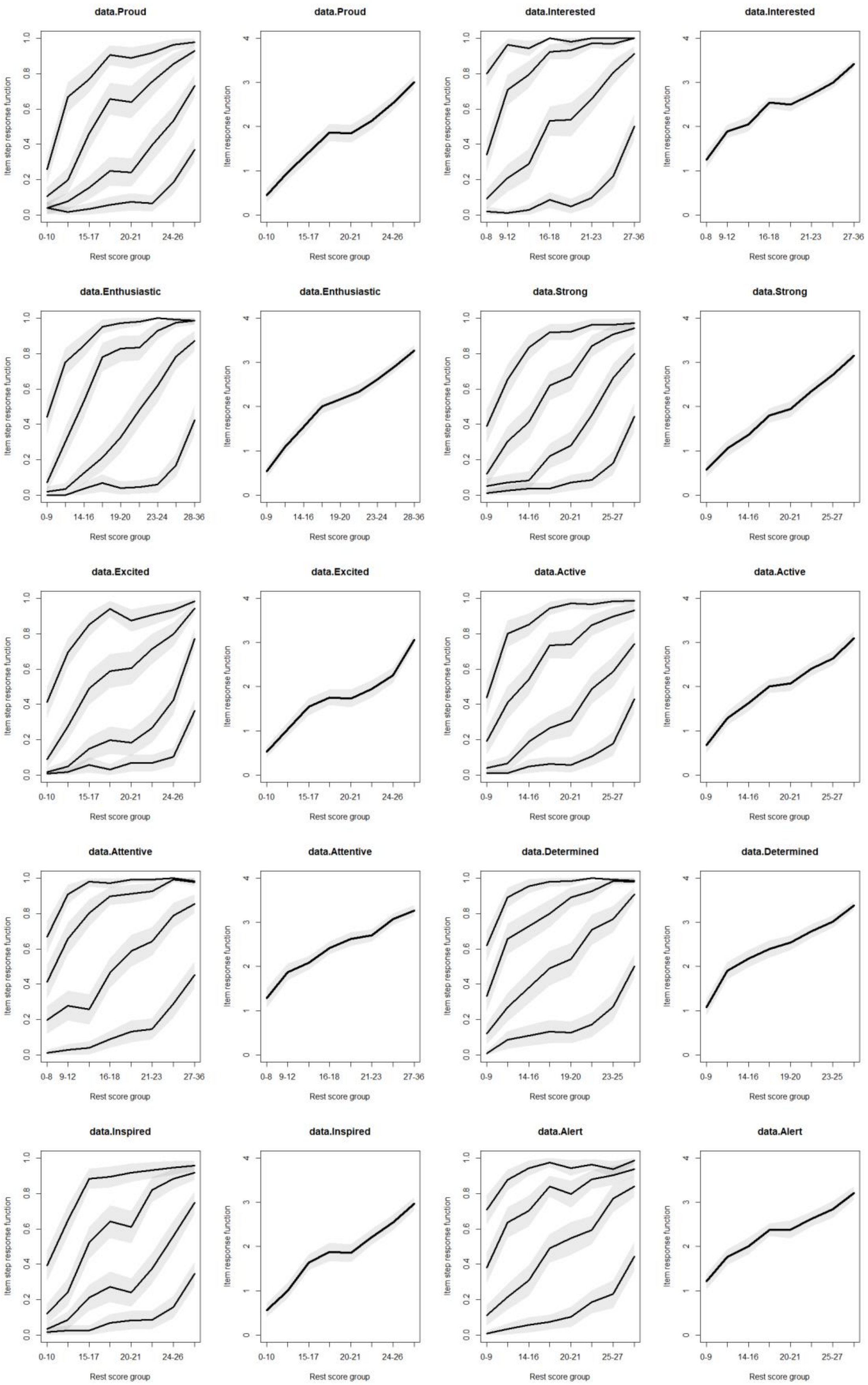

Figure S7: Monotonicity for Positive Affect.

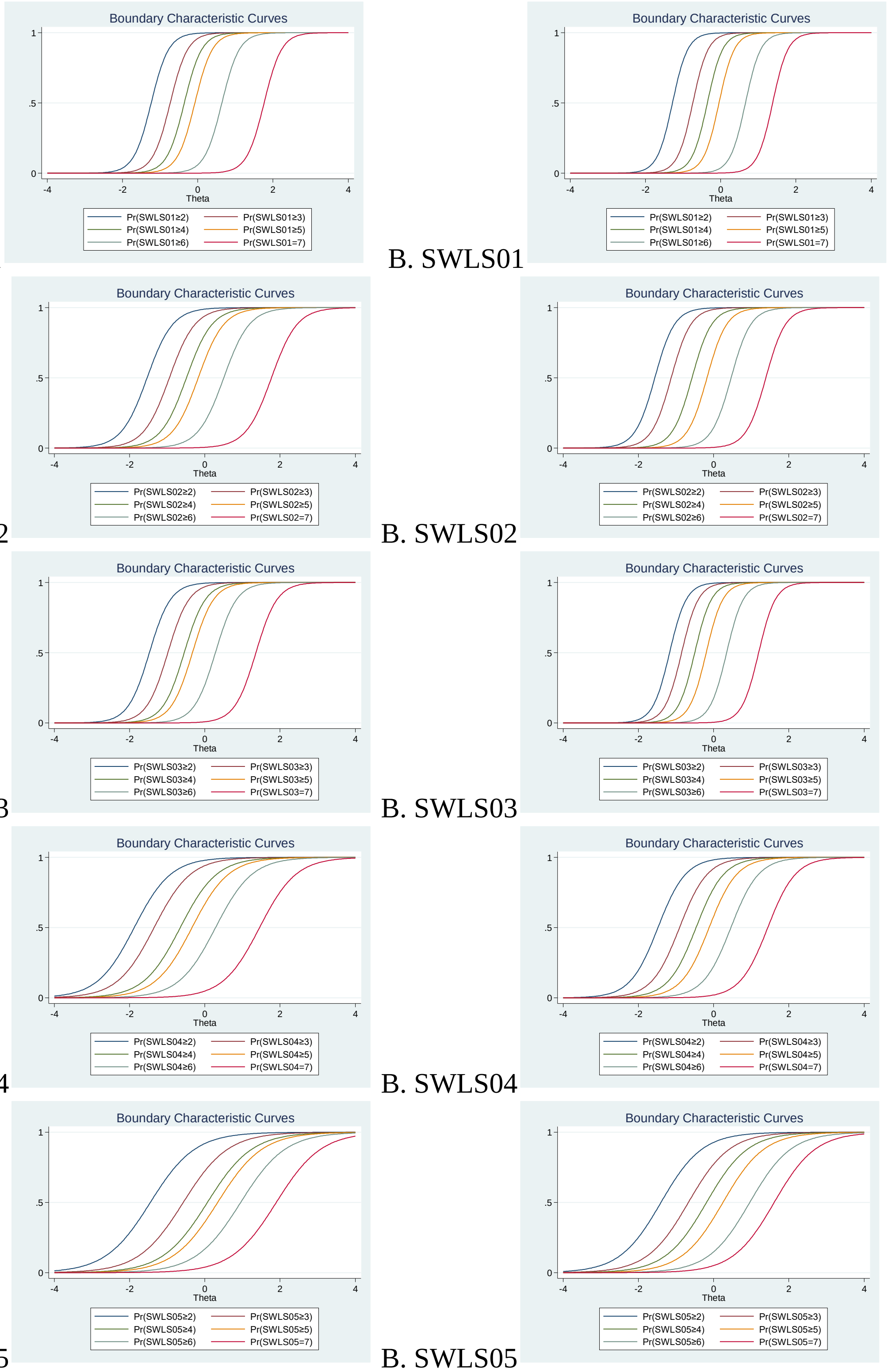

A. SWLS01 B. SWLS01

A. SWLS02 B. SWLS02

A. SWLS03 B. SWLS03

A. SWLS04 B. SWLS04

A. SWLS05 B. SWLS05

Figure S8: ICC, Uniform DIF regarding Satisfation with Life Scale (SWLS) for females (A) and males (B).

Note: SWLS01: “In most ways my life is close to my ideal”; SWLS02: “The conditions of my life are excellent”; SWLS03: “I am satisfied with my life”; SWLS04: “So far, I have gotten the important things I want in life”; and SWLS05: “If I could live my life over, I would change almost nothing”.

Table S1: Fit index of the models.

Fit Index	GRM	RSM	PCM
**Log-likelihood**	-3850.00	-3912.96	-3983.72
**AIC**	7770.01	7895.91	8029.43
**BIC**	7917.52	8043.43	8160.09

Table S2a: The standardized residuals among items in all measures in the study.

Satisfaction with Life Scale (SWLS)	SWLS01	SWLS02	SWLS03	SWLS04	SWLS05					
SWLS01	-	0.197	0.226	0.173	0.175					
SWLS02	-116.788	-	0.303	0.202	0.113					
SWLS03	-153.208	-275.858	-	0.189	0.188					
SWLS04	-90.138	-122.163	-107.068	-	0.15					
SWLS05	-91.694	-38.517	-105.779	67.289	-					
Harmony in Life Scale (HILS)	HILS01	HILS02	HILS03	HILS04	HILS05					
HILS01	-	0.465	0.432	0.26	0.233					
HILS02	-649.934	-	-1.538	0.371	0.176					
HILS03	-560.64	-7097.65	-	0.159	0.199					
HILS04	-203.166	-412.807	-76.203	-	0.151					
HILS05	-162.233	-93.185	-118.687	68.732	-					
Negative Affect (NA)	Distressed	Upset	Guilty	Afraid	Hostile	Irritable	Ashamed	Nervous	Jittery	Scared
Distressed	-	0.16	0.095	0.092	0.078	0.132	0.097	0.105	0.085	0.101
Upset	102.496	-	0.094	0.126	0.102	0.115	0.095	0.097	0.104	0.116
Guilty	-36.187	-35.432	-	0.062	0.072	0.066	0.148	0.079	0.052	0.082
Afraid	-33.65	-63.975	-15.445	-	0.083	0.123	0.065	0.058	0.093	0.157
Hostile	24.593	41.751	-20.689	-27.801	-	0.136	0.078	0.101	0.067	0.089
Irritable	70.107	52.758	-17.555	-60.839	73.67	-	0.076	0.128	0.107	0.142
Ashamed	-37.389	-35.726	87.325	-16.992	-24.1	-23.343	-	0.079	0.08	0.081
Nervous	-43.991	-37.636	-24.944	13.473	-40.713	-65.208	-24.704	-	0.132	0.076
Jittery	-28.808	-43.627	-10.679	-34.511	-17.722	-45.551	-25.557	69.79	-	0.078
Scared	-40.95	-53.499	-26.863	98.078	-31.736	-80.596	-25.946	23.21	-24.238	-
Positive Affect (PA)	Interested	Enthusiastic	Proud	Alert	Inspired	Determined	Attentive	Active	Excited	Strong
Interested	-	0.13	0.126	0.107	0.118	0.105	0.097	0.081	0.1	0.137
Enthusiastic	67.466	-	0.16	0.101	0.135	0.119	0.128	0.099	0.129	0.163
Proud	-63.757	-102.538	-	0.092	0.159	0.113	0.105	0.1	0.118	0.132
Alert	45.856	-40.573	-33.542	-	0.085	0.108	0.207	0.106	0.133	0.093
Inspired	-55.555	72.499	-100.539	-29.109	-	0.111	0.134	0.139	0.135	0.118
Determined	-43.894	-56.563	-50.73	46.766	49.318	-	0.115	0.125	0.121	0.09
Attentive	37.685	-65.9	-44.202	171.247	-71.846	53.103	-	0.096	0.153	0.141
Active	-25.958	-39.366	-39.796	-44.742	-77.298	-62.922	-36.704	-	0.1	0.105
Excited	-39.726	66.768	-55.644	-70.492	72.62	-58.824	-93.766	-40.22	-	0.121
Strong	-74.857	-105.833	70.028	-34.863	-55.243	32.239	-79.56	43.904	-58.51	-

Note: For the local dependence (LD) type, the upper diagonal elements represent the standardized residuals in the form of signed Cramers V coefficients. SWLS01: “In most ways my life is close to my ideal”; SWLS02: “The conditions of my life are excellent”; SWLS03: “I am satisfied with my life”; SWLS04: “So far, I have gotten the important things I want in life”; and SWLS05: “If I could live my life over, I would change almost nothing”; HILS01: “My lifestyle allows me to be in harmony”; HILS02: “Most aspects of my life are in balance”; HILS03: “I am in harmony”; HILS04: “I accept the various conditions of my life”; and HILS05: “I fit in well with my surroundings”.

Table S2b: The degrees of freedom and *p*-values for standardized residuals among items in all measures in the study.

Satisfaction with Life Scale (SWLS)	SWLS01	SWLS02	SWLS03	SWLS04	SWLS05					
SWLS01	-	0	0	0	0.000					
SWLS02	36	-	0	0	0.356					
SWLS03	36	36	-	0	0.000					
SWLS04	36	36	36	-	0.001					
SWLS05	36	36	36	36	-					
Harmony in Life Scale (HILS)	HILS01	HILS02	HILS03	HILS04	HILS05					
HILS01	-	0	0	0	0.000					
HILS02	36	-	0	0	0.000					
HILS03	36	36	-	0	0.000					
HILS04	36	36	36	-	0.001					
HILS05	36	36	36	36	-					
Negative Affect (NA)	Distressed	Upset	Guilty	Afraid	Hostile	Irritable	Ashamed	Nervous	Jittery	Scared
Distressed	-	0	0.003	0.006	0.077	0.000	0.002	0.000	0.025	0.001
Upset	16	-	0.003	0.000	0.000	0.000	0.003	0.002	0.000	0.000
Guilty	16	16	-	0.492	0.191	0.351	0.000	0.071	0.829	0.043
Afraid	16	16	16.000	-	0.033	0.000	0.386	0.638	0.005	0.000
Hostile	16	16	16.000	16.000	-	0.000	0.087	0.001	0.340	0.011
Irritable	16	16	16.000	16.000	16.000	-	0.105	0.000	0.000	0.000
Ashamed	16	16	16.000	16.000	16.000	16.000	-	0.075	0.061	0.055
Nervous	16	16	16.000	16.000	16.000	16.000	16.000	-	0.000	0.108
Jittery	16	16	16.000	16.000	16.000	16.000	16.000	16.000	-	0.084
Scared	16	16	16.000	16.000	16.000	16.000	16.000	16.000	16.000	-
Positive Affect (PA)	Interested	Enthusiastic	Proud	Alert	Inspired	Determined	Attentive	Active	Excited	Strong
Interested	-	0	0	0.000	0.000	0	0.002	0.055	0.001	0.000
Enthusiastic	16	-	0	0.001	0.000	0	0.000	0.001	0.000	0.000
Proud	16	16	-	0.006	0.000	0	0.000	0.001	0.000	0.000
Alert	16	16	16	-	0.023	0	0.000	0.000	0.000	0.004
Inspired	16	16	16	16.000	-	0	0.000	0.000	0.000	0.000
Determined	16	16	16	16.000	16.000	-	0.000	0.000	0.000	0.009
Attentive	16	16	16	16.000	16.000	16	-	0.002	0.000	0.000
Active	16	16	16	16.000	16.000	16	16.000	-	0.001	0.000
Excited	16	16	16	16.000	16.000	16	16.000	16.000	-	0.000
Strong	16	16	16	16.000	16.000	16	16.000	16.000	16.000	-

Note: Lower diagonal = degrees of freedom. Upper diagonal = *p*-values. SWLS01: “In most ways my life is close to my ideal”; SWLS02: “The conditions of my life are excellent”; SWLS03: “I am satisfied with my life”; SWLS04: “So far, I have gotten the important things I want in life”; and SWLS05: “If I could live my life over, I would change almost nothing”; HILS01: “My lifestyle allows me to be in harmony”; HILS02: “Most aspects of my life are in balance”; HILS03: “I am in harmony”; HILS04: “I accept the various conditions of my life”; and HILS05: “I fit in well with my surroundings”.

Table S3: Monotonicity for each item in all measures in the study.

Item	H	#ac	#vi	#vi/#ac	maxvi	sum	sum/#ac	zmax	#zsig	crit
SWLS01	0.71	28	0	0	0	0	0	0	0	0
SWLS02	0.68	31	0	0	0	0	0	0	0	0
SWLS03	0.70	36	0	0	0	0	0	0	0	0
SWLS04	0.63	36	0	0	0	0	0	0	0	0
SWLS05	0.58	36	0	0	0	0	0	0	0	0
HILS01	0.73	36	0	0	0	0	0	0	0	0
HILS02	0.76	32	0	0	0	0	0	0	0	0
HILS03	0.76	30	0	0	0	0	0	0	0	0
HILS04	0.66	30	0	0	0	0	0	0	0	0
HILS05	0.64	33	0	0	0	0	0	0	0	0
Interested	0.51	84	1	0.01	0.03	0.03	4.00E-04	0.76	0	1
Enthusiastic	0.55	123	0	0.00	0.00	0.00	0.00E+00	0.00	0	0
Proud	0.49	112	0	0.00	0.00	0.00	0.00E+00	0.00	0	0
Alert	0.42	112	2	0.02	0.04	0.08	7.00E-04	1.10	0	10
Inspired	0.47	112	1	0.01	0.03	0.03	3.00E-04	0.36	0	0
Determined	0.48	107	0	0.00	0.00	0.00	0.00E+00	0.00	0	0
Attentive	0.46	106	0	0.00	0.00	0.00	0.00E+00	0.00	0	0
Active	0.47	112	0	0.00	0.00	0.00	0.00E+00	0.00	0	0
Excited	0.49	112	2	0.02	0.07	0.10	9.00E-04	1.39	0	11
Strong	0.48	112	0	0.00	0.00	0.00	0.00E+00	0.00	0	0
Distressed	0.58	84	0	0	0	0	0	0	0	0
Upset	0.57	61	0	0	0	0	0	0	0	0
Guilty	0.50	61	0	0	0	0	0	0	0	0
Afriad	0.58	61	0	0	0	0	0	0	0	0
Hostile	0.47	76	0	0	0	0	0	0	0	0
Irritable	0.51	72	0	0	0	0	0	0	0	0
Ashamed	0.53	83	0	0	0	0	0	0	0	0
Nervous	0.56	79	0	0	0	0	0	0	0	0
Jittery	0.44	100	0	0	0	0	0	0	0	0
Scared	0.58	55	0	0	0	0	0	0	0	0

Note: SWLS01: "In most ways my life is close to my ideal"; SWLS02: "The conditions of my life are excellent"; SWLS03: "I am satisfied with my life"; SWLS04: "So far, I have gotten the important things I want in life"; and SWLS05: "If I could live my life over, I would change almost nothing"; HILS01: "My lifestyle allows me to be in harmony"; HILS02: "Most aspects of my life are in balance"; HILS03: "I am in harmony"; HILS04: "I accept the various conditions of my life"; and HILS05: "I fit in well with my surroundings".

Table S4: Indices of item fit and Benjamini-Hochberg criterion for *p*-value adjustment for items in the study.

Satisfaction with Life Scale (SWLS)	S_X2	df.S_X2	RMSEA.S_X2	*P*.S_X2	Rank	(i/m)Q
SWLS01	36.876	40	0.000	0.612	3	0.06
SWLS02	39.321	47	0.000	0.779	5	0.1
SWLS03	53.773	51	0.010	0.369	2	0.04
**SWLS04**	**85.597**	**64**	**0.026**	**0.037**	1	0.02
SWLS05	69.668	75	0.000	0.652	4	0.08
Harmony in Life Scale (HILS)						
**HILS01**	**55.083**	**37**	**0.031**	**0.028**	1	**0.02**
HILS02	34.871	37	0.000	0.569	5	0.1
HILS03	48.018	37	0.024	0.106	4	0.08
HILS04	74.361	60	0.022	0.100	3	0.06
**HILS05**	**79.838**	**59**	**0.027**	**0.037**	2	**0.04**
Positive Affect (PA)						
Interested	48.603	64	0.000	0.923	9	0.09
Enthusiastic	63.559	69	0.000	0.662	8	0.08
Proud	55.343	77	0.000	0.970	10	0.1
Alert	95.653	88	0.009	0.271	3	0.03
Inspired	83.750	81	0.006	0.395	6	0.06
Determined	79.884	83	0.000	0.577	7	0.07
Attentive	105.177	84	0.016	0.059	1	0.01
Active	85.751	82	0.007	0.367	4	0.04
Excited	84.985	74	0.012	0.180	2	0.02
Strong	81.732	79	0.006	0.394	5	0.05
Negative Affect (NA)						
Distressed	53.566	63	0.000	0.796	9	0.09
Upset	71.579	67	0.008	0.328	7	0.07
Guilty	74.106	72	0.005	0.409	8	0.08
Afraid	47.267	59	0.000	0.864	10	0.1
Hostile	76.210	69	0.010	0.258	5	0.05
Irritable	91.596	78	0.013	0.139	4	0.04
Ashamed	71.677	65	0.010	0.266	6	0.06
**Nervous**	**92.460**	**68**	**0.019**	**0.026**	2	0.02
Jittery	98.931	79	0.016	0.064	3	0.03
**Scared**	**85.639**	**57**	**0.022**	**0.008**	1	**0.01**

Note: bold type is significant. i = individual p-value's rank, m = total number of tests, Q = false discovery rate = .10. SWLS01: "In most ways my life is close to my ideal"; SWLS02: "The conditions of my life are excellent"; SWLS03: "I am satisfied with my life"; SWLS04: "So far, I have gotten the important things I want in life"; and SWLS05: "If I could live my life over, I would change almost nothing"; HILS01: "My lifestyle allows me to be in harmony"; HILS02: "Most aspects of my life are in balance"; HILS03: "I am in harmony"; HILS04: "I accept the various conditions of my life"; and HILS05: "I fit in well with my surroundings".
